# Leptin/Adiponectin Ratios Using Either Total Or High-Molecular-Weight Adiponectin as Biomarkers of Systemic Insulin Sensitivity in Normoglycemic Women

**DOI:** 10.1155/2017/9031079

**Published:** 2017-05-25

**Authors:** Carolina Bravo, Luis Rodrigo Cataldo, José Galgani, Javier Parada, José Luis Santos

**Affiliations:** ^1^Department of Nutrition, Diabetes and Metabolism, School of Medicine, Pontificia Universidad Católica de Chile, Santiago, Chile; ^2^Carrera de Nutrición y Dietética, Facultad de Medicina, Pontificia Universidad Católica de Chile, Santiago, Chile; ^3^School of Food Engineering, Universidad Austral de Chile, Valdivia, Chile

## Abstract

Plasma leptin/adiponectin ratio (LAR) is negatively associated with insulin sensitivity indexes. High-molecular-weight adiponectin (HMWA) was proposed as the most biologically active form of this insulin-sensitizing adipokine. There are no studies assessing the relative merits of leptin/HMWA ratio over LAR as a biomarker of systemic insulin sensitivity. A standard 2-hour oral glucose tolerance test (OGTT; 75 g of glucose) and a short minimal-model intravenous glucose tolerance test (IVGTT; 0.3 g/kg body weight) were performed in 58 Chilean normoglycemic women (age: 27 ± 6.3 years, BMI 23.6 ± 3.2 kg/m^2^). LAR was negatively associated with HOMA-S (*r* = −0.49; *p* < 0.0001), Matsuda-ISICOMP (*r* = −0.54; *p* < 0.0001), and the calculated sensitivity index (CSi) derived from IVGTT (*r* = −0.38; *p* = 0.007). In comparison to LAR, leptin/HMWA ratio did not increase neither the linear fit (*r*^2^) nor the magnitude of association with insulin sensitivity indexes (slope of multiple linear regression). The discriminatory capacity of both ratios to classify insulin-resistant versus insulin-sensitive subjects was similar for HOMA-S (*p* = 0.84), Matsuda-ISICOMP (*p* = 0.43), or CSi (*p* = 0.50). In conclusion, LAR showed consistent negative associations with different systemic insulin sensitivity indexes. The use of HMWA to generate leptin/HMWA ratio did not show any advantage over LAR as a biomarker of systemic insulin sensitivity in normoglycemic women.

## 1. Introduction

Diabetes is a group of metabolic diseases characterized by hyperglycemia resulting from defects in insulin secretion, action, or both [1]. Among patients with diabetes, type 2 diabetes mellitus (T2DM) is the most common disease, representing about 90% of all DM patients [2–4].

Insulin is secreted by pancreatic *β*-cells in response to increased circulating glucose, being also modulated by other multiple signals (hormones, monoamines, amino acids, nonesterified fatty acids, and incretins). One of the main functions of insulin is the maintenance of glucose homeostasis, which is achieved by allowing glucose uptake through GLUT4 in insulin-sensitive tissues (muscle and adipose tissues) and by inhibiting hepatic gluconeogenesis and glycogen degradation. In obesity, a relative hyperinsulinemic status has been described [5], which may compensate for the tissue-defective action of this hormone, and thus, maintain euglycemia.

Leptin is an adipokine mainly produced by adipose tissue that circulates in plasma in concentrations that are proportional to body fat. It has been reported that leptin acts as a signal of energy reserves in the central nervous system regulating energy intake. In the congenital leptin deficiency, the lack of this hormone generates hyperphagia and massive obesity, which is reversed following peripheral administration of recombinant human leptin. On the other hand, patients with congenital generalized lipodystrophy, a rare genetic disorder characterized by extreme leanness and very low levels of circulating leptin, display high-insulin resistance and premature diabetes. In these patients, the administration of human recombinant leptin also results in a remarkable improvement of glycemic control, indicating the insulin-sensitizing effects of this hormone in such disease [6]. Conversely, patients with common obesity show high-circulating leptin and insulin levels. The administration of human recombinant leptin to these patients does not seem to reduce intake or body fat, indicating a resistance to leptin action in multifactorial obesity.

Adiponectin is an adipokine produced by adipose tissue that circulates in high concentrations in plasma (representing up to 0.01% of total plasma proteins) [7]. In humans, lower plasma adiponectin concentrations have been found in patients with insulin resistance conditions such as obesity, T2DM, and coronary artery disease, compared to control subjects. A recent study showed that individuals with high-plasma adiponectin levels have a substantially lower adjusted relative risk for developing T2DM [8, 9]. It has been proposed that adiponectin circulates in plasma as multimers of different sizes such as trimers and hexamers, or as a form known as high-molecular-weight adiponectin (HMWA) [10]. Different lines of evidence have shown that HMWA is likely to be the most bioactive form of this adipokine [11], possibly representing a more relevant indicator of insulin sensitivity than a total adiponectin [12, 13].

Different studies have assessed plasma leptin/adiponectin ratio (LAR) in relation to whole-body (HOMA-IR) or muscle (hyperinsulinemic-euglycemic clamp) insulin sensitivity [14]. However, there are no studies evaluating the influence of including HMWA instead of total adiponectin on the performance of leptin/adiponectin ratios as biomarkers of systemic insulin sensitivity assessed through oral or intravenous glucose tests. Herein, we assessed whether the ratio represents a better index than LAR as a biomarker of systemic insulin sensitivity in normoglycemic women.

## 2. Subjects and Methods

### 2.1. Study Design and Subjects

A cross-sectional study was performed in 58 Chilean normoglycemic woman volunteers, without family history of diabetes, aged 27 ± 6.3 years, and BMI of 23.6 ± 3 2 kg/m^2^ (mean ± SD) (Table 1). The volunteers visited the UC Centre of Clinical Research in three occasions one week apart approximately. In the first visit, biochemical and anthropometric measurements were carried out such as weight, height, BMI, glucose and fasting plasmatic insulin, total cholesterol, HDL cholesterol, triglycerides, and systolic and diastolic arterial pressure (Table 1). At this stage, subjects with diabetes, glucose intolerance, dyslipidemia, or in pregnancy were excluded from the study. In the second visit, participants were submitted to a standard 75 g oral glucose tolerance test (OGTT), after 8–12 hours of fasting and drawing of blood samples at times −15, −5, 15, 30, 60, 90, and 120 minutes after glucose ingestion, for the determination of plasma glucose and insulin levels. In fasting samples (−15 minutes), an aliquot was separated for measuring leptin, adiponectin, and HMWA. In the third visit, participants were submitted to a short minimal-model intravenous glucose tolerance test (IVGTT; 0.3 g/kg body weight) [15] to measure plasma glucose and insulin at times −15, −5, 2, 4, 6, 8, 10, 15, 20, 30, 40, and 50 minutes after glucose intravenous administration. All participants signed written informed consent, and the research protocol was approved by the Ethics Committee of the School of Medicine of the Pontificia Universidad Católica de Chile.

### 2.2. Biochemical Determinations

Plasma levels of insulin (*μ*U/mL) and glucose (mg/dL) were measured in the central laboratory of the Pontificia Universidad Católica de Chile by electrochemiluminescence and colorimetric methods, respectively. Leptin and adiponectin concentrations were measured by radioimmunoassay (RIA), while plasma concentrations of high-molecular-weight adiponectin (HMWA) were measured by ELISA (R&D Systems).

### 2.3. Insulin Sensitivity Indexes

The Matsuda-ISICOMP index was calculated using OGTT (http://mmatsuda.diabetes-smc.jp/english.html). The calculated sensitivity index (CSi) derived from minimal-model IVGTT (abbreviated minimal model) was calculated with insulin and glucose levels measured from minutes 10 to 50 (minutes 10, 15, 20, 30, 40, and 50) using the website http://webmet.pd.cnr.it/csi/ [15]. Finally, the HOMA-S index was calculated using the formula: 1/fasting insulin (*μ*UI/mL) × fasting glucose (mg/dL)/405). This index represents the inverse of the HOMA-IR index (HOMA-S = 1/HOMA-IR).

### 2.4. Statistical Analyses

Summary statistics are expressed as mean ± standard deviation and quartiles. We used the Pearson and Spearman correlation coefficients and multiple linear regression models to assess the association between study variables. Initially, correlation analyses between pairs of variables were carried out after logarithmic transformation in both axes. Considering a correlation coefficient of *r* = −0.61 between LAR and HOMA-S [16], a sample size of *n* = 58 provides a statistical power > 99% with a confidence of 99% to detect significant associations between measurements LAR with insulin sensitivity indexes in our study (Matsuda-ISICOMP and CSi). The present study is based on a nonrandom sample of healthy volunteers that is not representative of any specific population.

Regression analysis was carried out with insulin sensitivity indexes (HOMA-S, Matsuda, and CSi) as dependent variables and adipokine-derived variables (leptin, adiponectin, HMWA, LAR, and leptin/HMWA ratio) as independent variables. For each adipokine or adipokine-derived variable, we created indicator (dummy) variables using tertiles, for which the lowest third of LAR (or leptin/HMWA ratio) served as a reference group. Regression coefficients were estimated in crude analysis as well as in models adjusted by gender and BMI. As leptin, adiponectin, HMWA, LAR, and leptin/HMWA ratio are strongly related to BMI, we carried out an additional residual analysis from the regression lines. For this purpose, logarithmic transformation of leptin, adiponectin, HMWA, LAR, and leptin/HMWA ratio was performed to generate studentized residuals against BMI in multiple linear regression models. For each independent variable, the studentized residuals were classified into thirds (upper, middle, or lower) for their analysis of association with insulin sensitivity indexes as dependent variables.

Receiver operating characteristic (ROC) curve analysis was used to evaluate the discriminatory capacity of leptin, total adiponectin, HMWA, LAR, and leptin/HMWA ratio to classify insulin-resistant versus insulin-sensitive subjects defined by the median of HOMA-S, Matsuda-ISICOMP, or CSi. All statistical analyses were carried out with STATA 14.0 statistical software (http://www.stata.com).

## 3. Results

**Figure 1:**
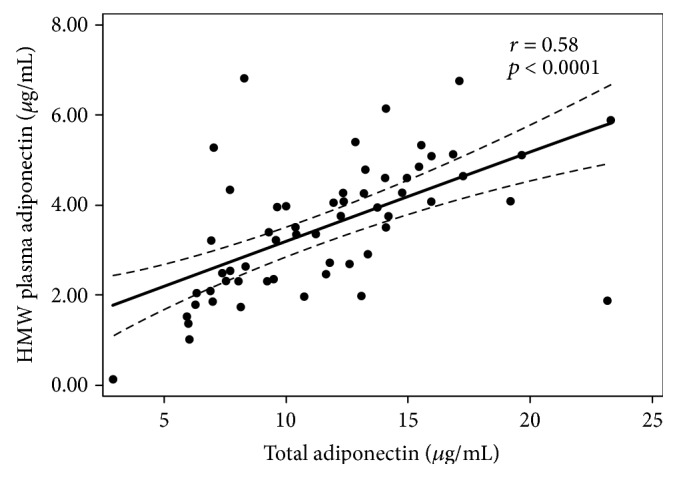
Correlation between high-molecular-weight adiponectin and total adiponectin in a cross-sectional sample of 58 Chilean normoglycemic women.

**Figure 2:**
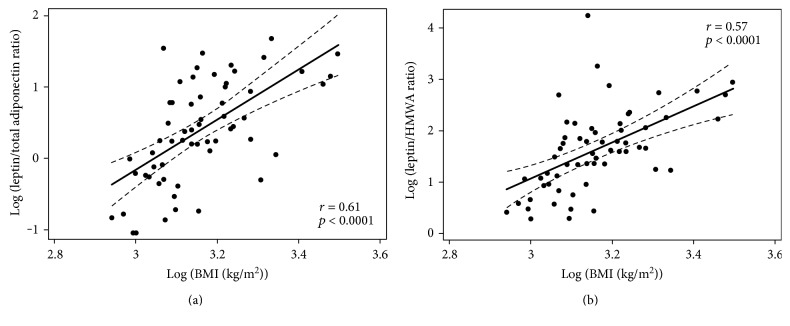
Association between leptin/total adiponectin ratio (LAR) and leptin/high-molecular-weight adiponectin (HMWA) ratio with body mass index (BMI) in a cross-sectional sample of 58 Chilean normoglycemic women.

**Figure 3:**
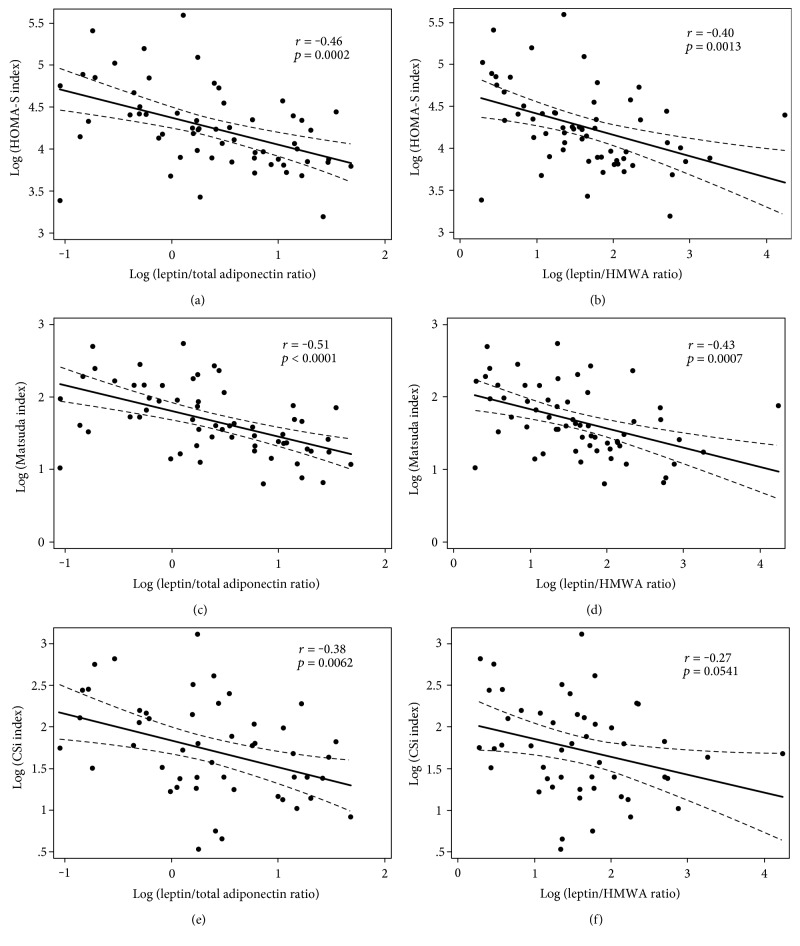
Associations between log (leptin/adiponectin ratio) and leptin/high-molecular-weight adiponectin (HMWA) ratio with sensitivity insulin indexes in a cross-sectional sample of 58 Chilean normoglycemic women.

Table 1 shows the summary statistics of the study group including age, weight, height, BMI, fasting insulin and glucose, total cholesterol, HDL cholesterol, triglycerides, and pressure (systolic and diastolic).  Table 2 shows the adipokine concentrations and adipokine-derived ratios (leptin, total adiponectin, HMWA, LAR, and leptin/HMWA ratio). Plasma concentrations of total adiponectin were positively correlated with those of HMWA (*r* = 0.58, *p* < 0.0001) (Figure 1). Insulin sensitivity indexes were not associated with BMI (Figure S1 available online at https://doi.org/10.1155/2017/9031079). The associations between leptin, total adiponectin, and HMWA with BMI are shown (Figure S2). LAR and leptin/HMWA ratio were positively associated with BMI (Figure 2). Although in different magnitude, LAR and leptin/HMWA ratio were associated with insulin sensitivity indexes (HOMA-S, Matsuda-ISICOMP, and CSi) (Figure 3). Leptin, adiponectin, and HMWA were also separately associated with insulin sensitivity indexes (Figure S3).

**Figure 4:**
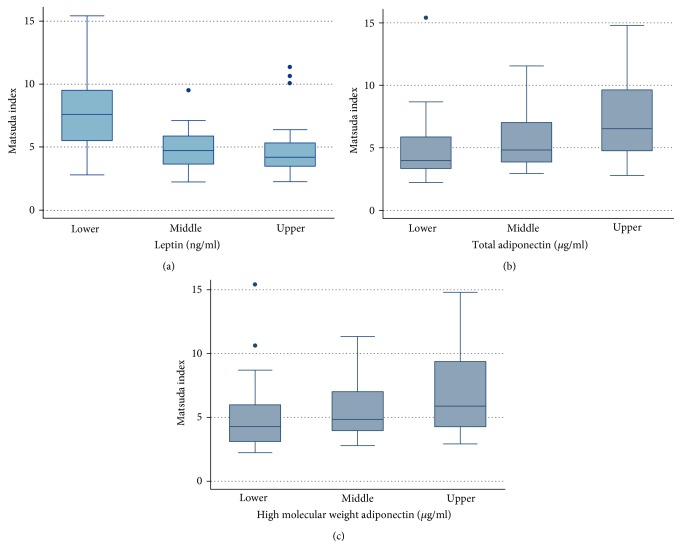
Box plots showing Matsuda-ISICOMP index by the thirds of leptin, adiponectin, and high-molecular-weight adiponectin in a cross-sectional sample of 58 Chilean normoglycemic women.

**Figure 5:**
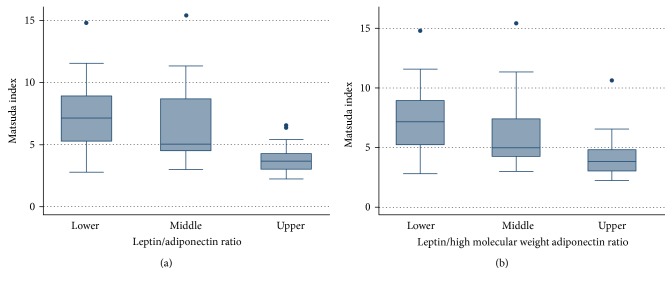
Association between leptin/total adiponectin and leptin/high-molecular-weight adiponectin ratio with Matsuda-ISICOMP index in a cross-sectional sample of 58 Chilean normoglycemic women.

One possible way to evaluate the performance of plasma leptin, total adiponectin, HMWA, LAR, and leptin/HMWA ratio with insulin sensitivity indexes is by comparing their slopes, where a greater slope means a stronger magnitude of association. However, it is not possible to carry out such direct comparison given that the ranges of variation of plasma leptin, total adiponectin, HMWA, LAR, and leptin/HMWA ratio are different, and therefore, the slopes are not comparable in a straightforward manner (Figure S3). Then, we divided the sample using two tertiles into three thirds of plasma leptin, total adiponectin, HMWA, LAR, and leptin/HMWA ratio. Using these categorical variables as dummy variables, we carried out linear regression analysis with Matsuda-ISICOMP (Figures 4 and 5), CSi, and HOMA-S as dependent variables (Figure S4). Matsuda-ISICOMP increased significantly across the thirds of adiponectin (total adiponectin and HMWA) (Figures 4(b) and 4(c)) while decreased significantly across the thirds of leptin (Figure 4(a)), LAR, and leptin/HMWA ratio (Figures 5(a) and 5(b)). Likewise, a similar pattern of associations has been found when comparing the upper versus lower thirds of these adipokines or its ratios with CSi and HOMA-S (Figures S4 and S5). Linear regression models relating indexes of insulin sensitivity with plasma leptin, total adiponectin, HMWA, LAR, and leptin/HMWA ratio showed a dose-response effect, which achieved statistical significance when comparing the upper and lower thirds of these variables (Figures 4 and 5, Figures S4 and S5). The use of leptin/HMWA ratio instead of LAR did not result in an increase in the magnitude of the association with indexes of insulin sensitivity (slopes of linear regression models), indicating that the behavior of both variables was similar in relation to insulin sensitivity indexes (see Table S1).

The associations between plasma adipokines and adipokine-derived ratios remained significant after adjustment for BMI in multiple linear regression models (Table S1). We additionally evaluated such associations using residual analysis, finding that associations between studentized residuals of leptin, total adiponectin, HMWA, LAR, and leptin/HMWA ratio (all log transformed) with respect to BMI showed significant associations with indexes of insulin sensitivity. The result of this analysis confirms that these variables show associations with HOMA-S, Matsuda, or CSi that remain significant even after adjustment for BMI (data not shown).

**Figure 6:**
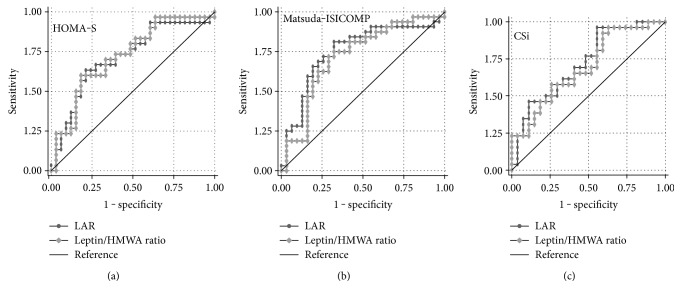
ROC curve analysis for discriminating insulin-sensitive versus insulin-resistant normoglycemic women using leptin/adiponectin (LAR) or leptin/HMWA ratio

Areas under the receiver operating characteristic (ROC) curve indicate that the discriminatory capacity of either LAR or leptin/HMWA ratio to classify insulin-resistant versus insulin-sensitive subjects was similar for HOMA-S (*p* = 0.84), Matsuda-ISICOMP (*p* = 0.43), or CSi (*p* = 0.50) (Figure 6). Likewise, no significant differences were found in ROC curve areas for plasma leptin, total adiponectin, and HMWA in relation to insulin sensitivity indexes (Table S2).

## 4. Discussion

The motivation of this study was double: on the one hand, to assess the performance of leptin, adiponectin and its ratios, LAR, and leptin/HMWA ratio, as useful biomarkers of systemic (whole body) insulin sensitivity. On the other hand, this study was conducted to gain insights on the physiologic significance of circulating adipokines in relation to insulin action and BMI. We have confirmed that plasma leptin levels were positively associated with BMI and negatively with insulin sensitivity indexes, whereas the opposite pattern of associations was observed for total adiponectin and HMWA. Such well-known associations emphasize the importance of these two adipokines connecting adipose tissue biology, obesity, and insulin sensitivity. In obesity, adipocytes secrete more leptin and less adiponectin, leading to the hypothesis that leptin/adiponectin ratios are useful biomarkers of adipocyte hypertrophy, insulin resistance, and cardiovascular risk. We have shown herein that LAR and leptin/HMWA ratio exhibited consistent negative associations with different systemic insulin sensitivity indexes derived from basal samples, as well as from oral or intravenous glucose loads.

**Figure 7:**
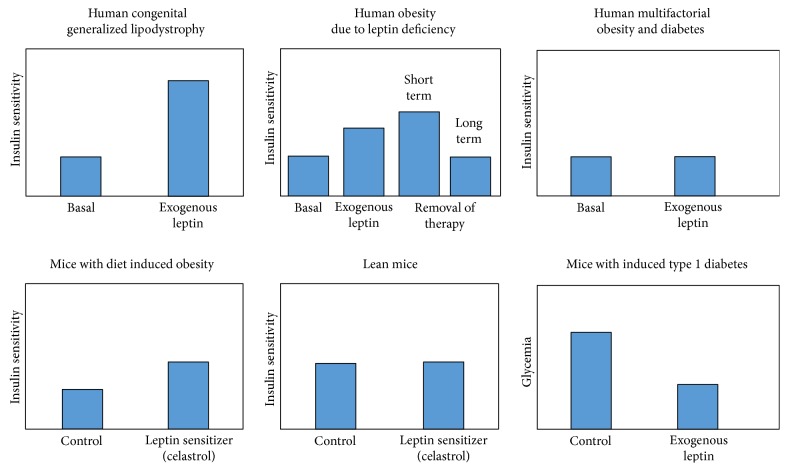
Effect of exogenous leptin administration on insulin sensitivity and diabetes-related traits in different contexts.

Multiple linear regression and residual analysis in our data showed that associations of leptin, LAR, and leptin/HMWA ratio with insulin sensitivity indexes remained significant even after adjustment by BMI, indicating that leptin and leptin-derived variables exert its effects beyond its association with adiposity. This observation has been repeatedly reported in human metabolic studies [16, 17]. We speculate that the predominant effect of leptin/adiponectin ratios (either LAR or leptin/HMWA ratio) on insulin sensitivity is apparently driven by leptin over adiponectin (or HMWA), given that the associations of leptin/adiponectin with HOMA-S, Matsuda-ISICOMP, or CSi display a negative slope (the same as what occurs with plasma leptin). As an attempt to conciliate our observations with the proposed actions of leptin described in the literature, Figure 7 shows published effects of exogenous leptin administration on insulin sensitivity and glycemic-related traits in different physiologic or physiopathologic contexts [6, 18, 19]. Recent reports have emphasized the concept that leptin has insulin-sensitizing effects, as it was demonstrated in patients affected with congenital generalized lipodystrophy and in mice treated with leptin sensitizers such as celastrol [6, 20]. Moreover, mice with induced type 1 diabetes treated with leptin are able to maintain euglycemia even in the absence of insulin, indicating the important actions of leptin in the hepatic endogenous production of glucose, possibly through mechanisms acting via central and autonomous nervous system, and/or through direct effects on pancreatic *α*-cells and glucagon production [21]. In rare cases of obesity due to leptin deficiency, the administration of human recombinant leptin leads to an apparent increase in insulin sensitivity, possibly associated with a rapid weight loss. Paradoxically, a further short-term increase in insulin sensitivity has been reported after withdrawal of leptin therapy (possibly due to the rapid increase in glucose-absorbing fat mass) [22]. However, this effect seems to be only transient since long-term follow-up after the withdrawal of leptin therapy should lead to obesity and to a reduction of insulin sensitivity. In T2DM patients with common multifactorial obesity, exogenous leptin administration does not seem to improve insulin sensitivity, possibly due to the resistance to leptin action [23–25], in a paradigm somehow concordant with what we have found in our observational study.

We have confirmed that both adiponectin and HMWA are positively associated with insulin sensitivity indexes and negatively with BMI, even in our group of normoglycemic, mainly normal-weight women with no family history of diabetes. Our results again support the important insulin-sensitizing effects described for this hormone [26–29]. In contrast, it is also worth noting that very high levels of insulin and adiponectin, combined with low leptin, have been described as a strong predictor of rare cases of severe insulin resistance due to mutations in the insulin receptor gene or anti-insulin receptor antibodies (type B insulin resistance) [30]. It has been reported that adiponectin circulates in three forms: low-, intermediate-, and high-molecular-weight multimers. It has been suggested that HMWA is the main source of the active form of this protein, which is related to metabolic syndrome [31–37]. However, some reports indicate that HMWA does not provide additional information over total adiponectin in relation to reference methods to measure insulin sensitivity in humans [38, 39]. Our study also supports the idea that HMWA does not improve the performance over total adiponectin in classifying insulin-resistant versus insulin-sensitive normoglycemic participants (Table S3).

We have not found publications evaluating the specific influence of including HMWA instead of total adiponectin on the performance of leptin/adiponectin ratios as biomarkers of systemic (whole body) insulin sensitivity. In this study, the use of HMWA instead of total adiponectin to generate the leptin/HMWA ratio did not show any advantage over LAR as a biomarker of insulin sensitivity in normoglycemic women using methods such as OGTT and IVGTT. The correct assessment of the relative advantages of LAR over leptin/HMWA ratio also deserves some discussion. We have compared both measurements in relation to insulin sensitivity indexes in terms of their performance in three statistical aspects: (1) We assessed the percentage of variation of insulin sensitivity indexes explained by either LAR or leptin/HMWA ratio through the Pearson correlation *r* or equivalently by the coefficient of determination *r*^2^. We have found that in all situations, the linear fit was greater using LAR over leptin/HMWA ratio. (2) We evaluated the magnitude of the association between leptin/adiponectin ratios with insulin sensitivity indexes, reflected in the slope relating such variables in a regression model. In this sense, we divided the sample using tertiles to achieve comparable measures of differences in insulin sensitivity indexes when participants were classified in thirds of either LAR or leptin/HMWA ratio (being the lowest third as references). Again, in all scenarios, we found that the slope relating LAR with insulin sensitivity indexes was of a higher magnitude than that obtained using leptin/HMWA ratio. Simple comparisons of *r*^2^ values or slopes in both (1) and (2) do not provide statistical straightforward evidence on the superiority of LAR over leptin/HMWA ratio, given that there is no available formal statistical test to assess the hypotheses involved in these comparisons. The lack of an appropriate statistical test is derived from the fact that correlation/regression models share the same dependent variable (insulin sensitivity indexes), and moreover, independent variables are strongly associated (LAR and leptin/HMWA ratio). In this context, (3) we also used receiver operating characteristic (ROC) curve analysis to compare the discriminatory capacity of LAR and leptin/HMWA ratio to distinguish insulin-resistant versus insulin-sensitive in normoglycemic women. Further studies comprising subjects with different glycemic status will be necessary to determine the cutoff points for insulin resistance based on plasma leptin and adiponectin levels. This procedure provides an adequate statistical support for the idea that using HMWA instead of total adiponectin to generate the leptin/HMWA ratio did not show any advantage over LAR as a biomarker of systemic insulin sensitivity in normoglycemic women.

One advantage of our study is the use of different methods to assess systemic insulin sensitivity, namely, fasting samples (to calculate HOMA-S), OGTT (to calculate Matsuda-ISICOMP index), or short IVGTT (to calculate CSi). The reference methods for estimating insulin sensitivity are the hyperinsulinemic-euglycemic clamp and the intravenous glucose tolerance test (IVGTT) based on Bergman's minimal model [40]. The hyperinsulinemic-euglycemic clamp is a costly and time-consuming approach that mainly reflects muscle insulin action. On the contrary, the IVGTT minimal model reflects whole-body insulin action, allowing the joint estimation of first-phase insulin secretion and insulin sensitivity in a single day. In our study, we aimed to focus on methods reflecting the systemic actions of insulin, ranging from surrogate markers based on fasting samples (HOMA-S), as well as from oral glucose load (OGTT) or intravenous glucose (IVGTT minimal model) tests. As indicated above, Finucane et al. [14] reported a high correlation between LAR with HOMA-S and M/I from the hyperinsulinemic-euglycemic clamp (representing mainly muscle insulin sensitivity). Furthermore, most studies assessing the relation of LAR with systemic insulin sensitivity have been conducted using HOMA-IR as a surrogate of insulin sensitivity, instead of more sophisticated methods derived from oral or intravenous glucose loads.

We carried out our study in a group of normoglycemic, mainly normal-weight women with no family history of diabetes. This relatively homogeneous and healthy group was selected because we wish to develop early biomarkers that precede states of dysglycemia and insulin resistance that can be used in ongoing population-based epidemiologic studies during childhood, adolescence, and young adulthood in Chile [41]. Although many surrogates of insulin sensitivity/resistance have been generated in the last years [42–45], LAR has the relative advantage that both leptin and adiponectin are stable measures that do not need to be measured in blood samples in fasted state. Although it has been reported a circadian rhythm for plasma leptin levels, such variations are not relevant for most hours in which blood samples are normally drawn in the clinic context. As a potential disadvantage for using LAR or leptin/HMWA ratio as a biomarker of insulin sensitivity, it is worth to mention that both leptin and adiponectin are not routinely available in most clinical biochemical laboratories. Among the weaknesses of the study, we can mention our relatively small sample size and the lack of information regarding other risk factor influencing insulin sensitivity such as diet or physical exercise.

## 5. Conclusion

We have confirmed in our group of normoglycemic women with no family history of diabetes that plasma leptin levels were positively associated with BMI and negatively with insulin sensitivity indexes, whereas the opposite was found both for adiponectin and HMWA. LAR showed consistent negative associations with different systemic insulin sensitivity indexes derived from basal samples, as well as, from oral or intravenous glucose loads. The use of HMWA instead of total adiponectin to generate the leptin/HMWA ratio did not show any advantage over LAR as a biomarker of insulin sensitivity in normoglycemic women. Therefore, LAR can be considered as an informative adipokine-related variable reflecting the role of adipose tissue in insulin biology, as well as an adequate biomarker of insulin sensitivity for monitoring future risk of T2DM in population-based epidemiologic studies.

## Supplementary Material

Supplemental Figure 1. Association between insulin sensitivity indexes with body mass index (BMI) in a cross-sectional sample of 58 Chilean normoglycaemic women. Supplemental Figure 2. Association between leptin, total adiponectin and high molecular weight adiponectin with body mass index (BMI) in a cross-sectional sample of 58 Chilean normoglycaemic women. HMWA: High Molecular Weight Adiponectin. Supplemental Figure 3. Associations between log of Leptin, Total Adiponectin and High Molecular Weight Adiponectin ratio with sensitivity insulin indexes in a cross-sectional sample of 58 Chilean normoglycaemic women. HMWA: High Molecular Weight Adiponectin. Supplemental Figure 4. Box plots showing thirds of leptin, total adiponectin and high molecular weight adiponectin with insulin sensitivity indexes (HOMA-S and CSi) in a cross-sectional sample of 58 Chilean normoglycaemic women. Supplemental Figure 5. Box plots showing thirds of leptin/total adiponectin and leptin/high molecular weight adiponectin with insulin sensitivity indexes (HOMA-S and CSi) index in a cross-sectional sample of 58 Chilean normoglycaemic women. Supplemental Table 1. Association between insulin sensitivity indexes with plasma leptin, total adiponectin, HMW-adiponectin and leptin/adiponectin ratios #. Supplemental Table 2. ROC curve analysis to evaluate the discriminatory capacity of leptin, total adiponectin, HMWA, LAR and leptin/HMWA ratio in relation to insulin sensitivity indexes.

## Figures and Tables

**Table 1:** Anthropometric and biochemical characteristics of Chilean normoglycemic women.

*n* = 58	Mean ± SD
Age (years)	26.9 ± 6.3
Weight (kg)	60.2 ± 9.2
Height (m)	1.6 ± 0.05
BMI (kg/m^2^)	23.6 ± 3.1
Fasting glucose (mg/dL)	79.7 ± 5.3
Fasting insulin (*μ*IU/mL)	8.4 ± 3.6
Total cholesterol (mg/dL)	173.1 ± 35.1
HDL cholesterol (mg/dL)	63.1 ± 14.7
Triglycerides (mg/dL)	106.7 ± 60.8
Systolic arterial pressure (mmHg)	111.8 ± 9.8
Diastolic blood pressure (mmHg)	70.5 ± 8.1

**Table 2:** Adipokine concentrations of participants of Chilean normoglycemic women.

*n* = 58	Mean ± SD	Quartiles 25% 50% 75%
Leptin (ng/mL)	18.4 ± 9.3	10.5
		16.5
		24.7

Total adiponectin (*μ*g/mL)	23.1 ± 8.3	7.9
		11.5
		14.1

Leptin/adiponectin ratio (LAR)	0.9 ± 0.6	0.9
		1.5
		2.8

HMWA (*μ*g/mL)	3.5 ± 1.4	2.4
		3.4
		4.4

Leptin/HMWA ratio	7.3 ± 9.5	3.0
		5.1
		8.0

HMWA: high-molecular-weight adiponectin.
