# Supplementary material for: Leptin/Adiponectin Ratios Using Either Total Or High-Molecular-Weight Adiponectin as Biomarkers of Systemic Insulin Sensitivity in Normoglycemic Women
Source: J Diabetes Res. 2017 May 25;2017:9031079. doi: 10.1155/2017/9031079 (PMC5463152; doi:10.1155/2017/9031079)
Supplement: Supplementary file 1 — Supplemental Figure 1. Association between insulin sensitivity indexes with body mass index (BMI) in a cross-sectional sample of 58 Chilean normoglycaemic women. Supplemental Figure 2. Association between leptin, total adiponectin and high molecular weight adiponectin with body mass index (BMI) in a cross-sectional sample of 58 Chilean normoglycaemic women. HMWA: High Molecular Weight Adiponectin. Supplemental Figure 3. Associations between log of Leptin, Total Adiponectin and High Molecular Weight Adiponectin ratio with sensitivity insulin indexes in a cross-sectional sample of 58 Chilean normoglycaemic women. HMWA: High Molecular Weight Adiponectin. Supplemental Figure 4. Box plots showing thirds of leptin, total adiponectin and high molecular weight adiponectin with insulin sensitivity indexes (HOMA-S and CSi) in a cross-sectional sample of 58 Chilean normoglycaemic women. Supplemental Figure 5. Box plots showing thirds of leptin/total adiponectin and leptin/high molecular weight adiponectin with insulin sensitivity indexes (HOMA-S and CSi) index in a cross-sectional sample of 58 Chilean normoglycaemic women. Supplemental Table 1. Association between insulin sensitivity indexes with plasma leptin, total adiponectin, HMW-adiponectin and leptin/adiponectin ratios #. Supplemental Table 2. ROC curve analysis to evaluate the discriminatory capacity of leptin, total adiponectin, HMWA, LAR and leptin/HMWA ratio in relation to insulin sensitivity indexes. [file 9031079.f1.pptx]

## Slide 1
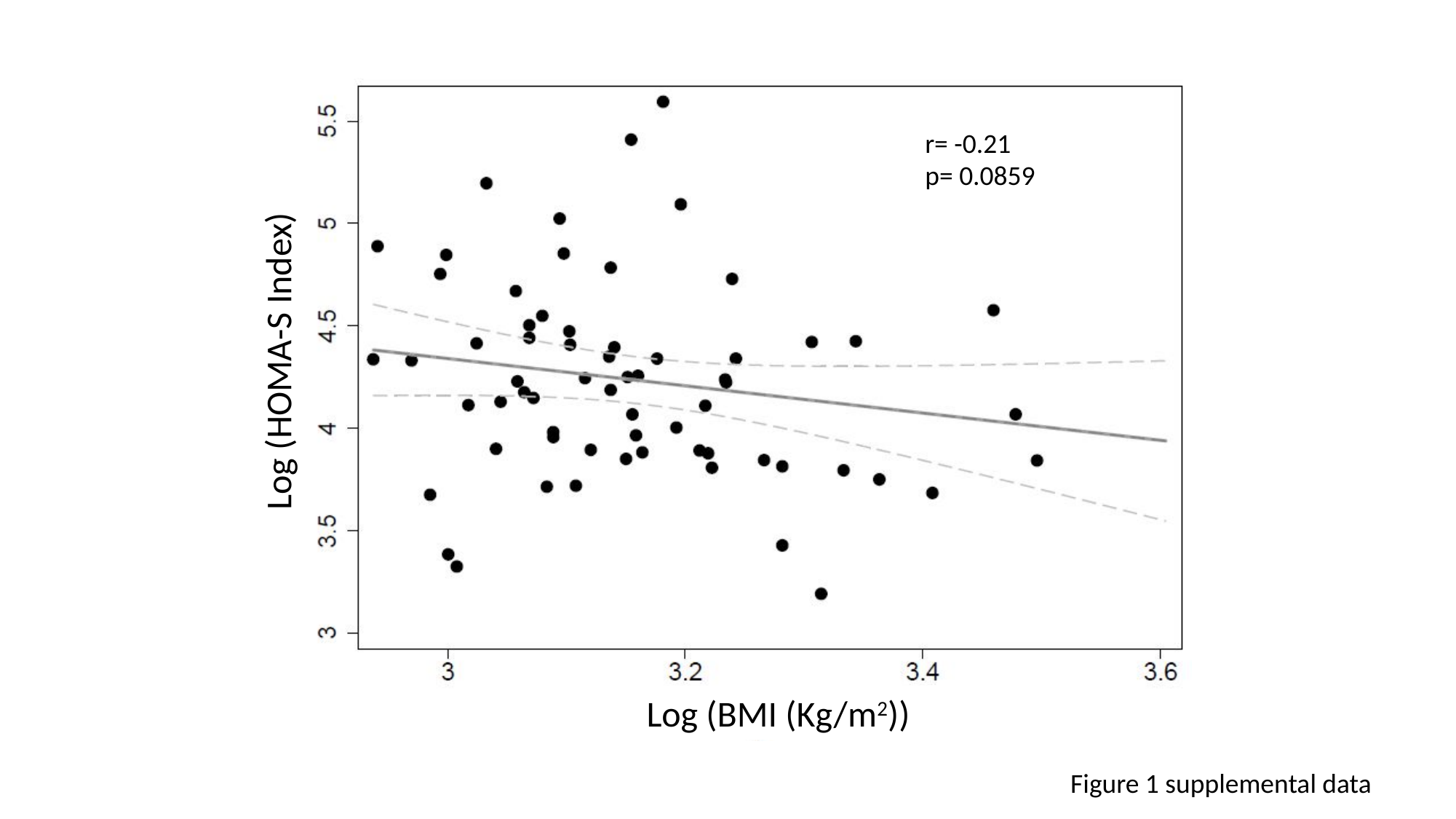

r= -0.21
p= 0.0859
 Log (HOMA-S Index)
Log (BMI (Kg/m2))
Figure 1 supplemental data

## Slide 2
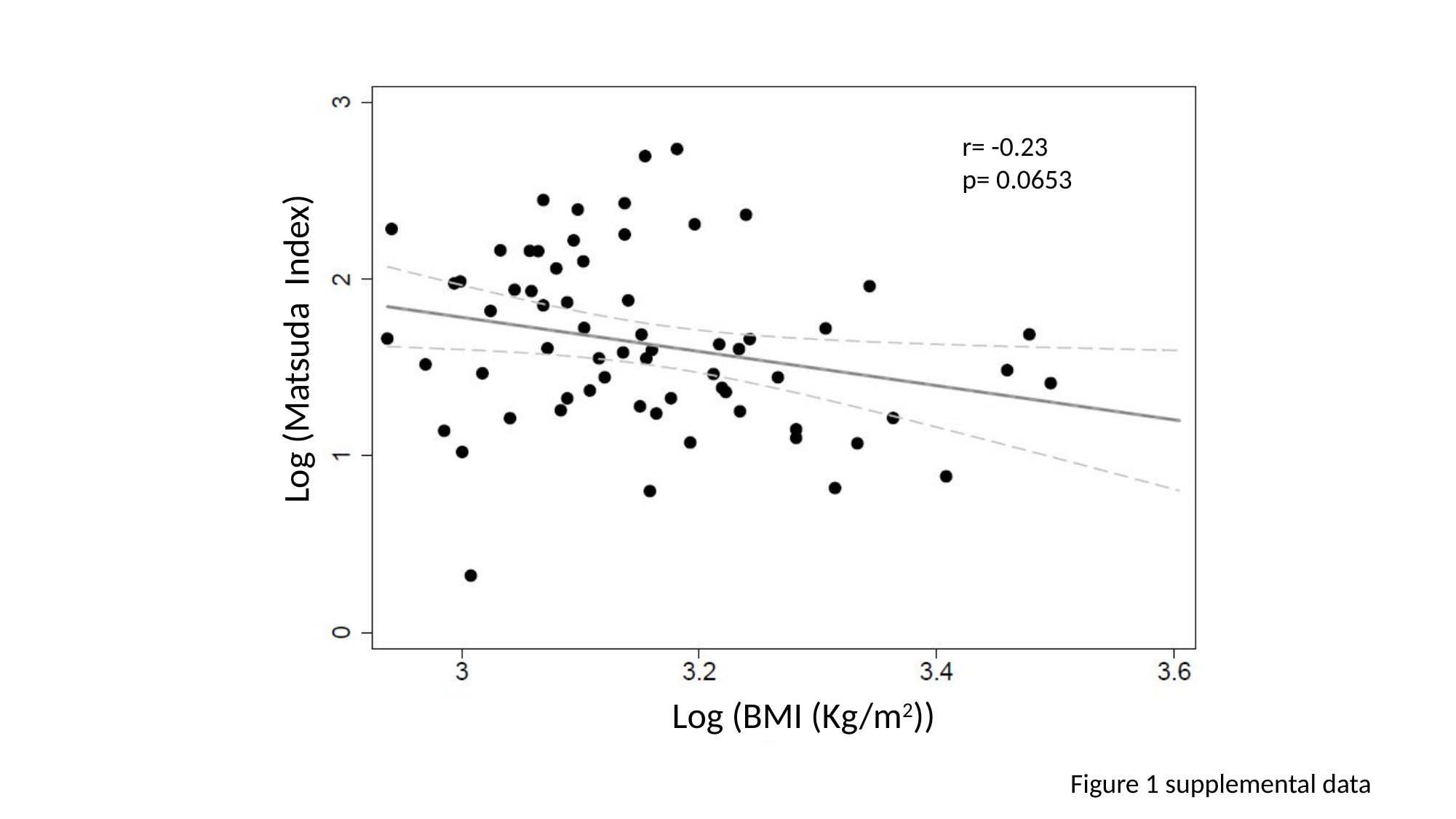

r= -0.23
p= 0.0653
 Log (Matsuda Index)
Log (BMI (Kg/m2))
Figure 1 supplemental data

## Slide 3
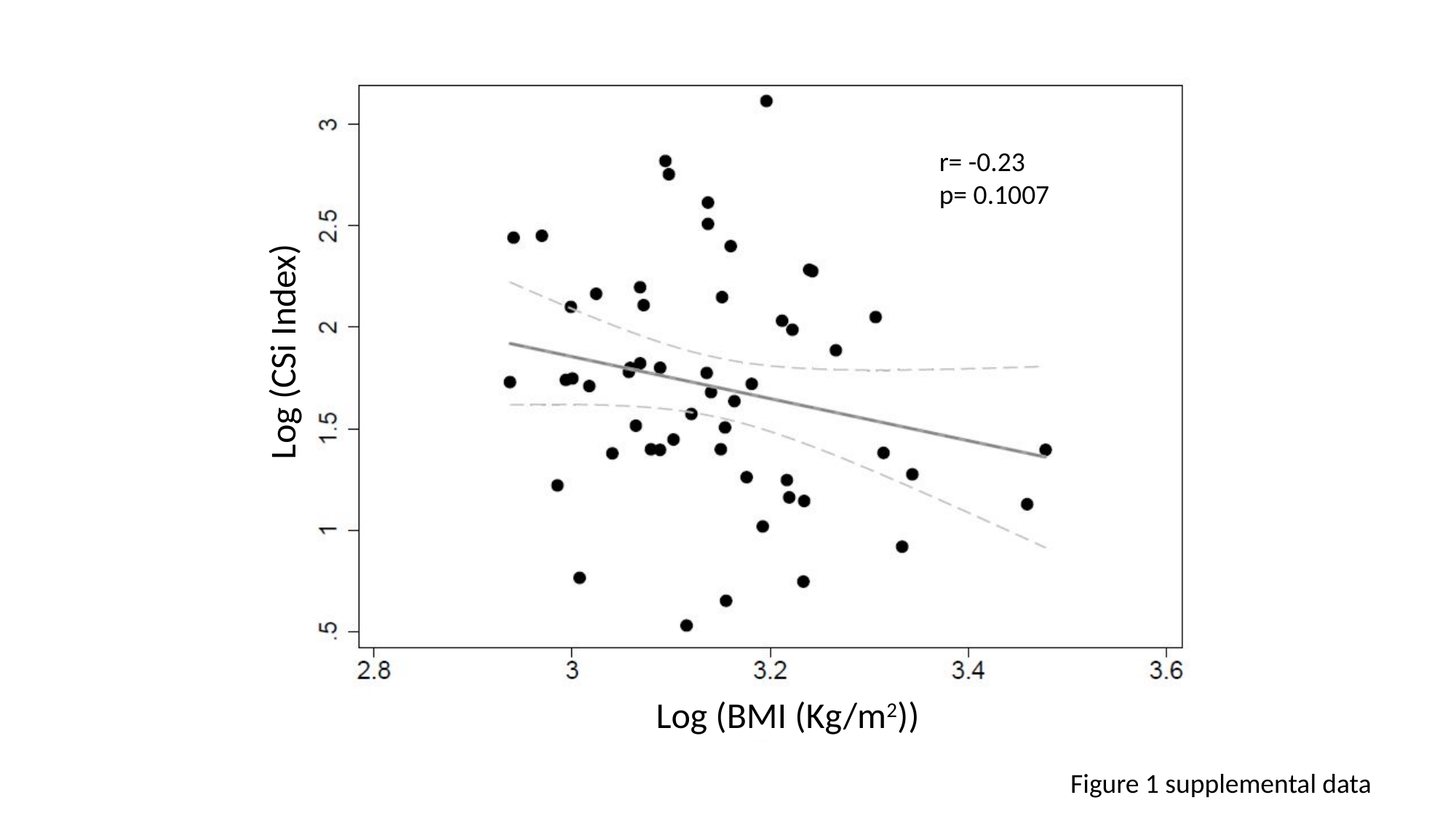

r= -0.23
p= 0.1007
 Log (CSi Index)
Log (BMI (Kg/m2))
Figure 1 supplemental data
