# Supplementary material for: Leptin/Adiponectin Ratios Using Either Total Or High-Molecular-Weight Adiponectin as Biomarkers of Systemic Insulin Sensitivity in Normoglycemic Women
Source: J Diabetes Res. 2017 May 25;2017:9031079. doi: 10.1155/2017/9031079 (PMC5463152; doi:10.1155/2017/9031079)
Supplement: Supplementary file 2 [file 9031079.f2.pptx]

## Slide 1
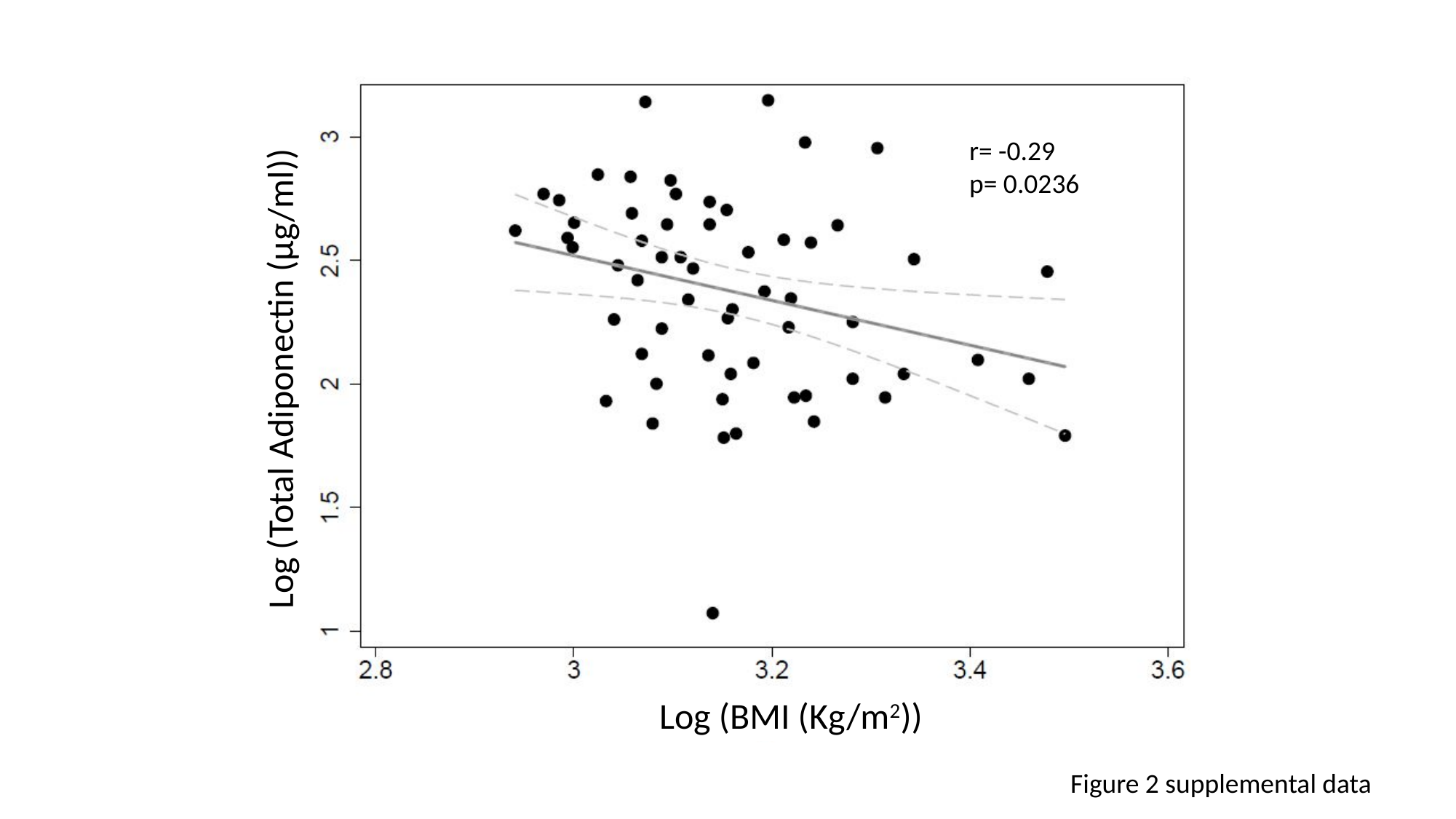

r= -0.29
p= 0.0236
Log (Total Adiponectin (µg/ml))
Log (BMI (Kg/m2))
Figure 2 supplemental data

## Slide 2
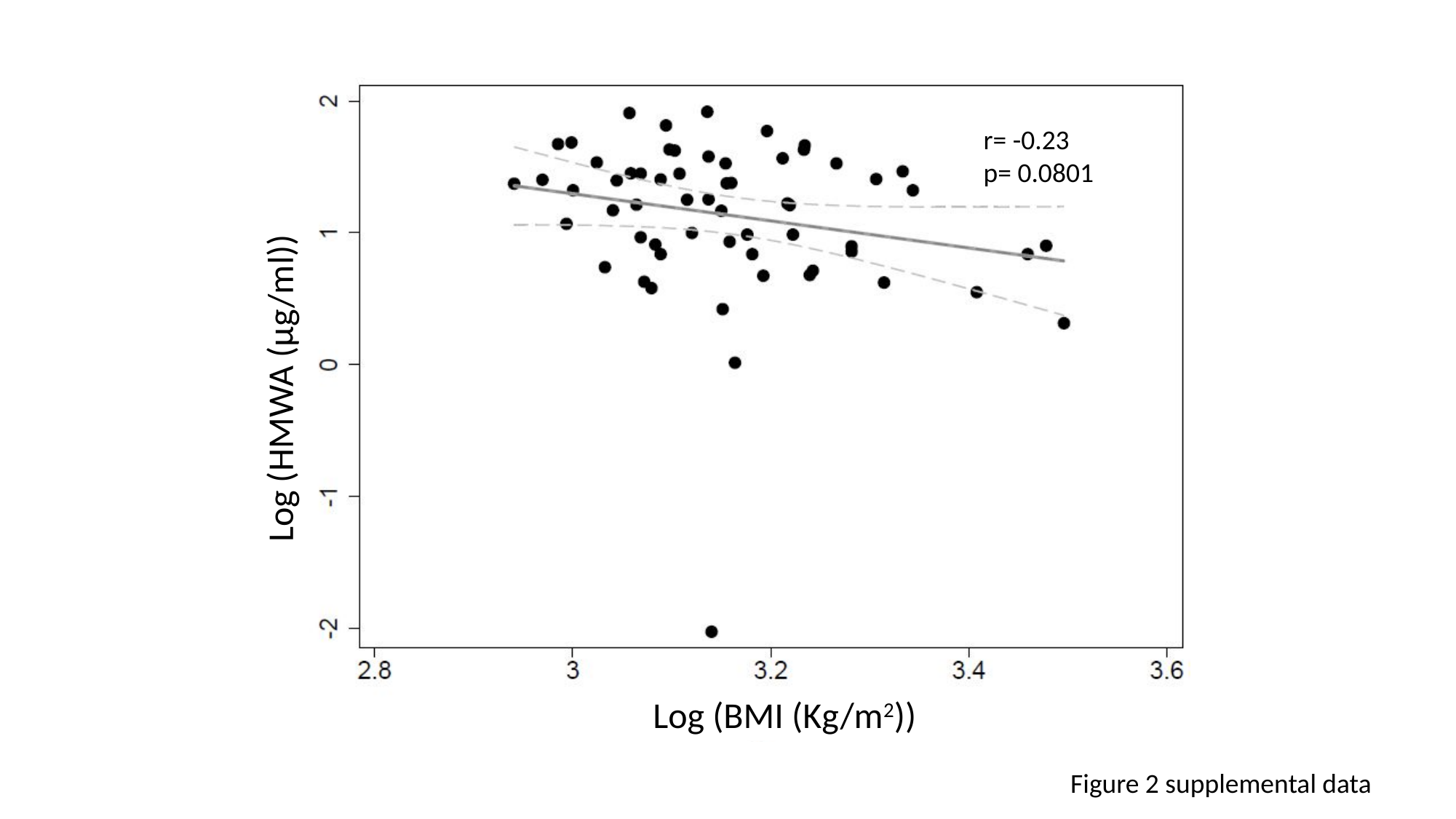

r= -0.23
p= 0.0801
Log (HMWA (µg/ml))
Log (BMI (Kg/m2))
Figure 2 supplemental data

## Slide 3
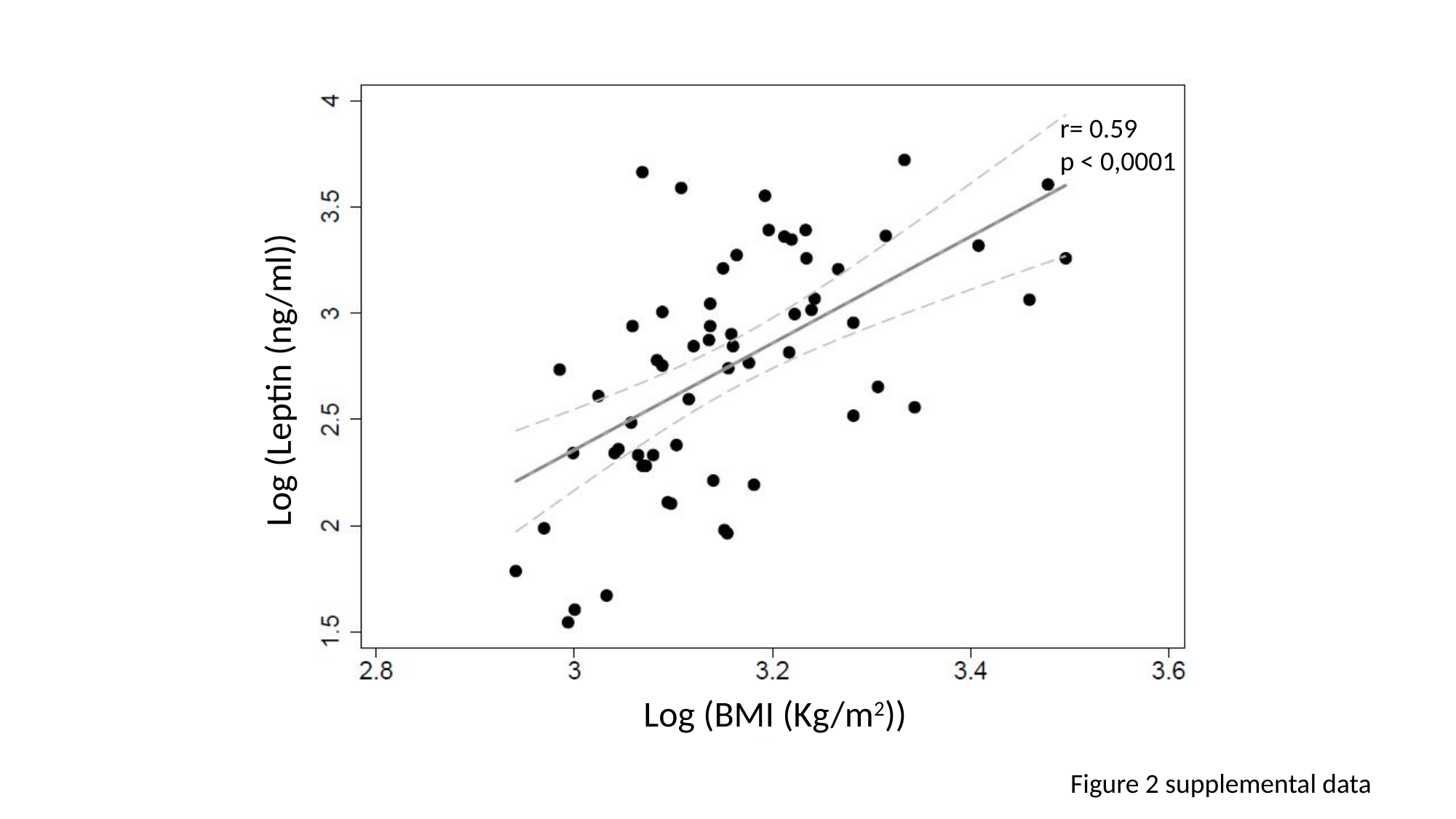

r= 0.59
p < 0,0001
Log (Leptin (ng/ml))
Log (BMI (Kg/m2))
Figure 2 supplemental data
