# Supplementary material for: Leptin/Adiponectin Ratios Using Either Total Or High-Molecular-Weight Adiponectin as Biomarkers of Systemic Insulin Sensitivity in Normoglycemic Women
Source: J Diabetes Res. 2017 May 25;2017:9031079. doi: 10.1155/2017/9031079 (PMC5463152; doi:10.1155/2017/9031079)
Supplement: Supplementary file 3 [file 9031079.f3.pptx]

## Slide 1
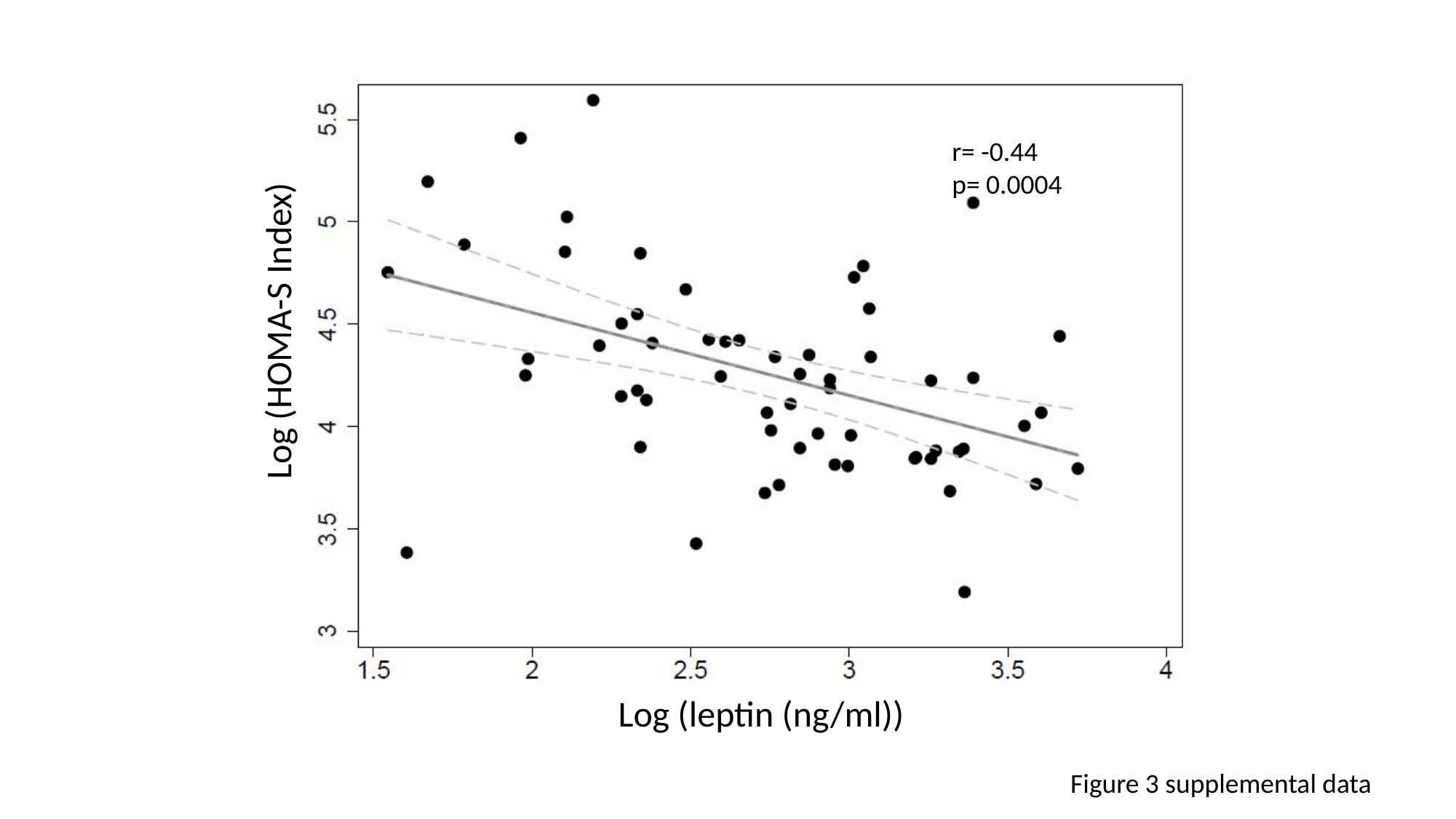

r= -0.44
p= 0.0004
 Log (HOMA-S Index)
Log (leptin (ng/ml))
Figure 3 supplemental data

## Slide 2
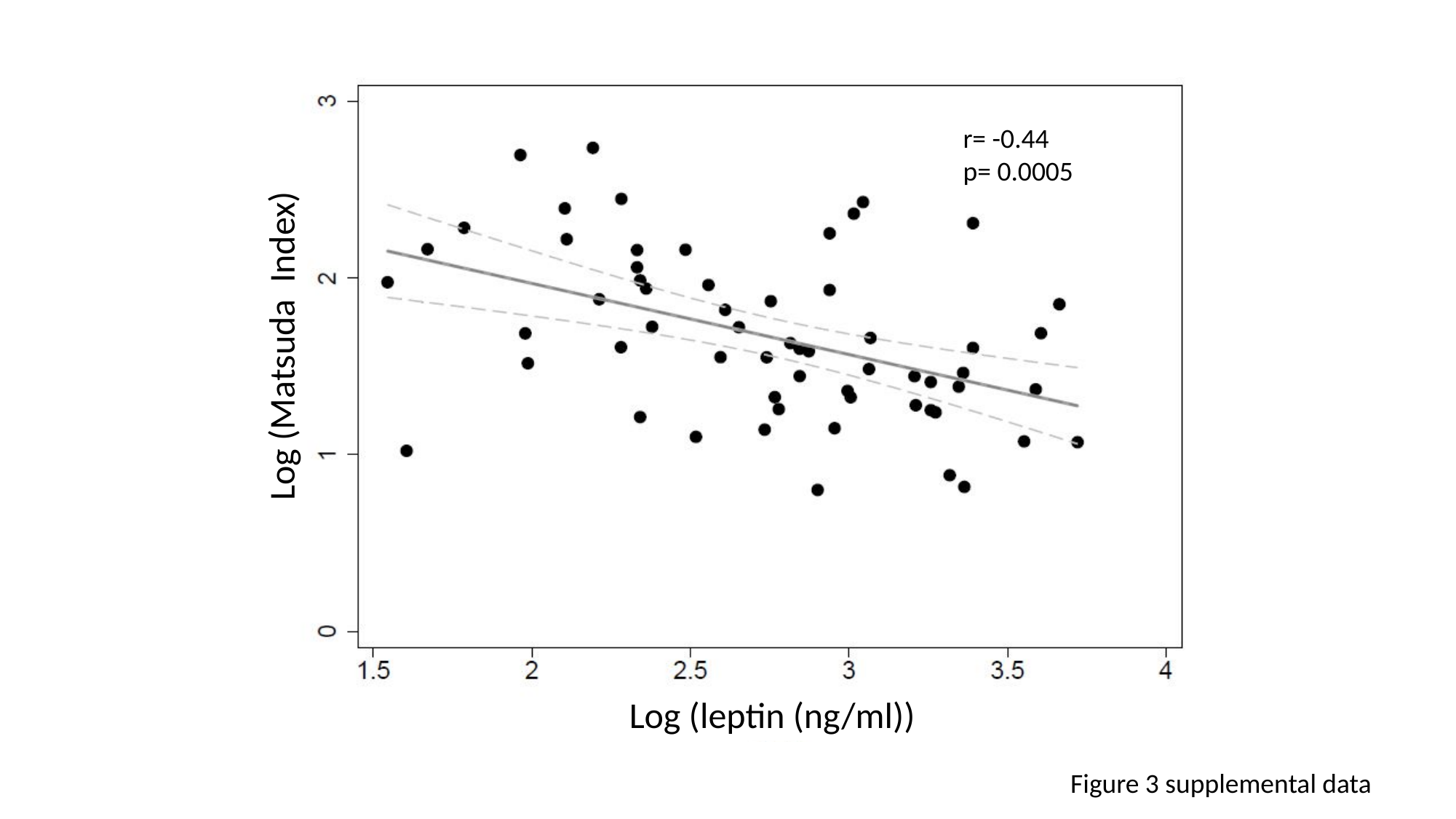

r= -0.44
p= 0.0005
 Log (Matsuda Index)
Log (leptin (ng/ml))
Figure 3 supplemental data

## Slide 3
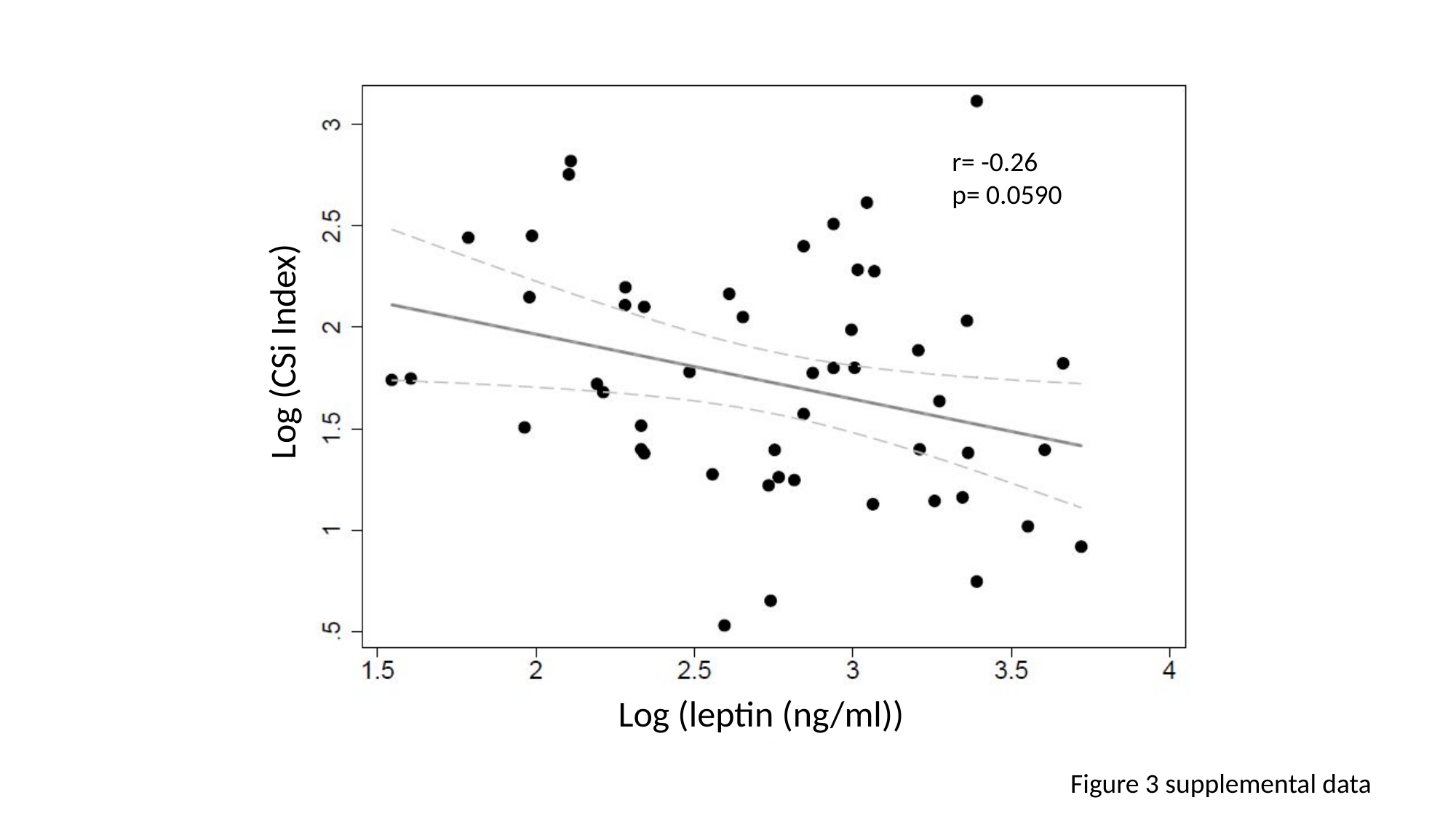

r= -0.26
p= 0.0590
 Log (CSi Index)
Log (leptin (ng/ml))
Figure 3 supplemental data

## Slide 4
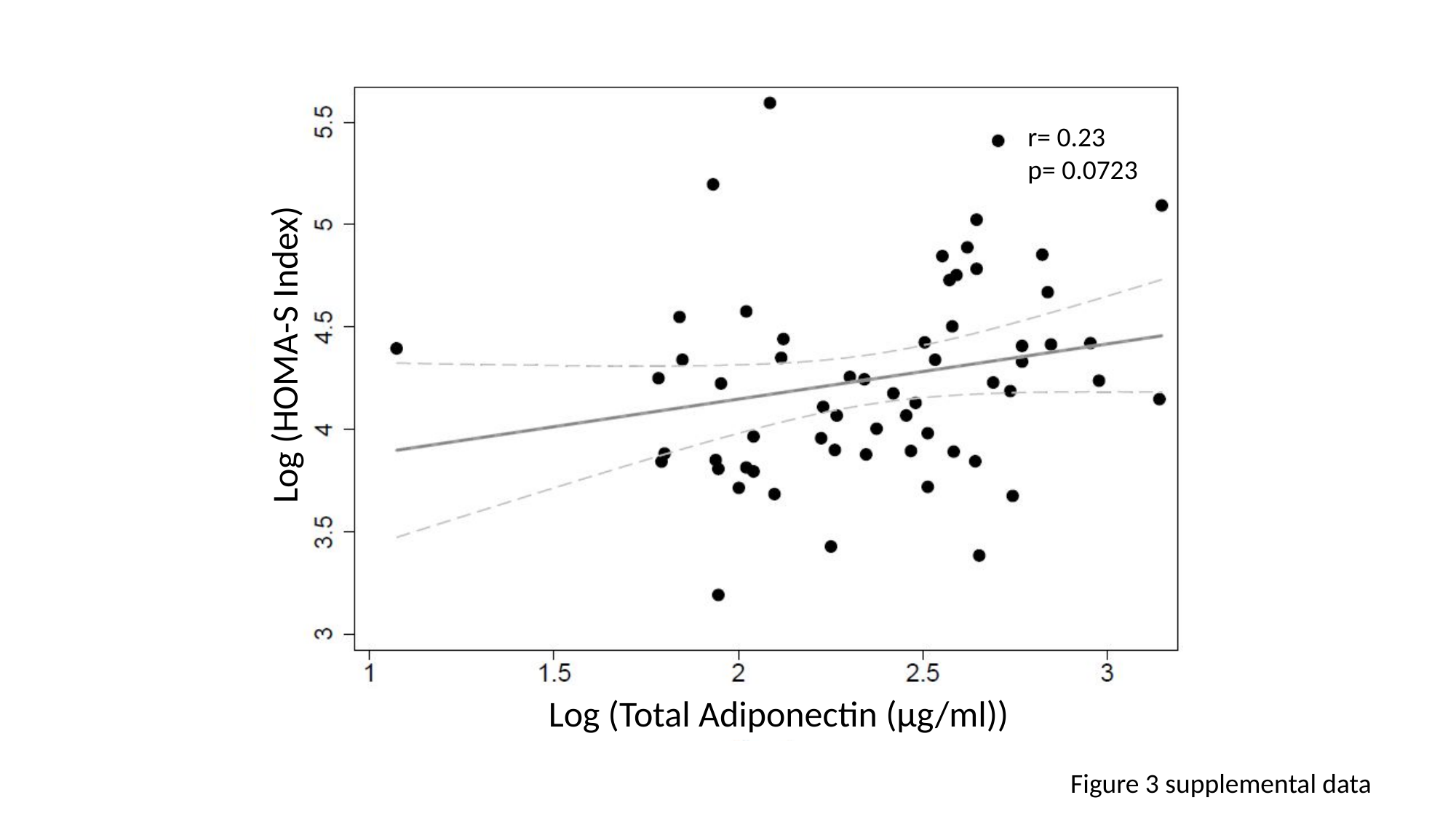

r= 0.23
p= 0.0723
 Log (HOMA-S Index)
Log (Total Adiponectin (µg/ml))
Figure 3 supplemental data

## Slide 5
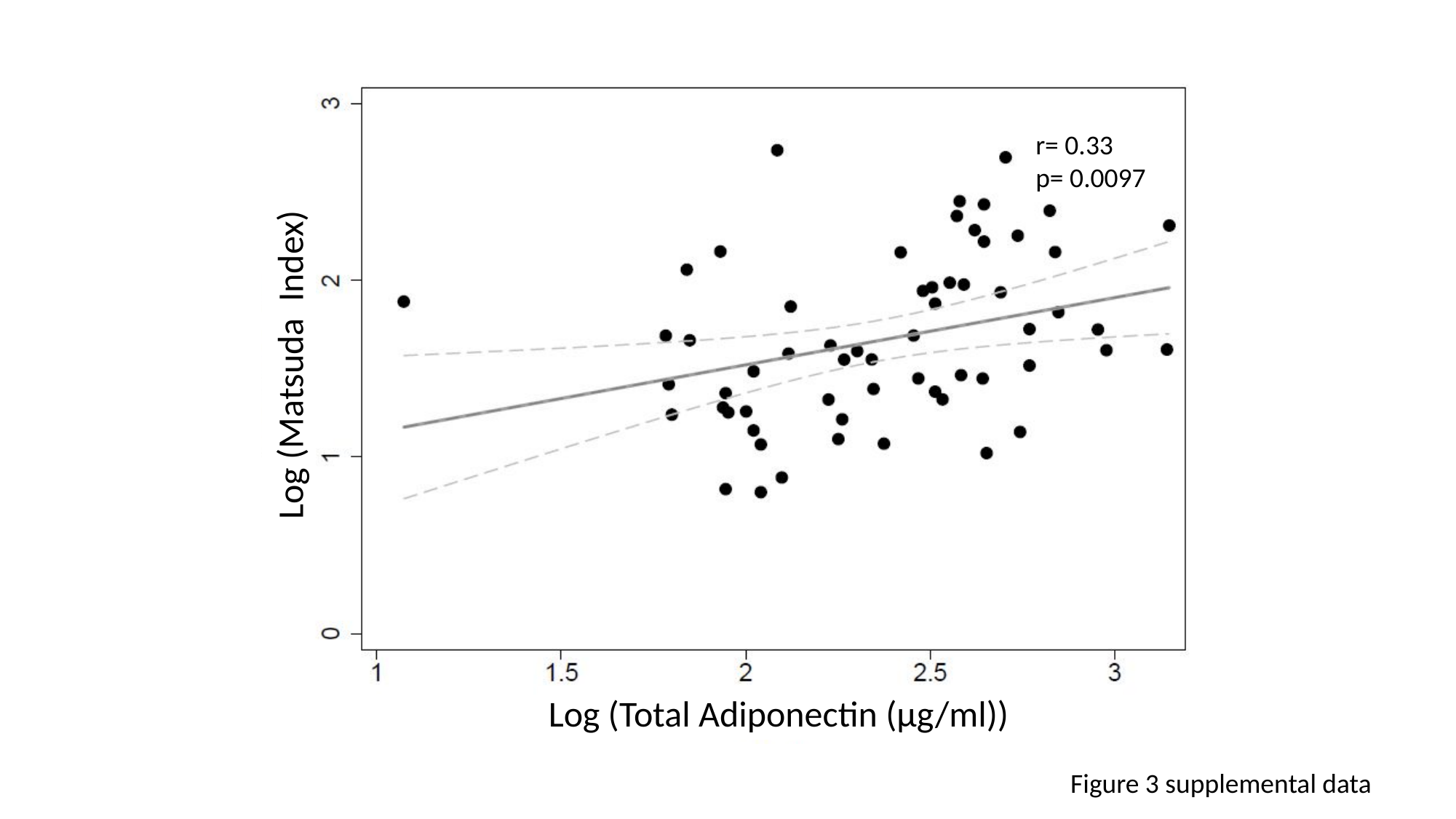

r= 0.33
p= 0.0097
 Log (Matsuda Index)
Log (Total Adiponectin (µg/ml))
Figure 3 supplemental data

## Slide 6
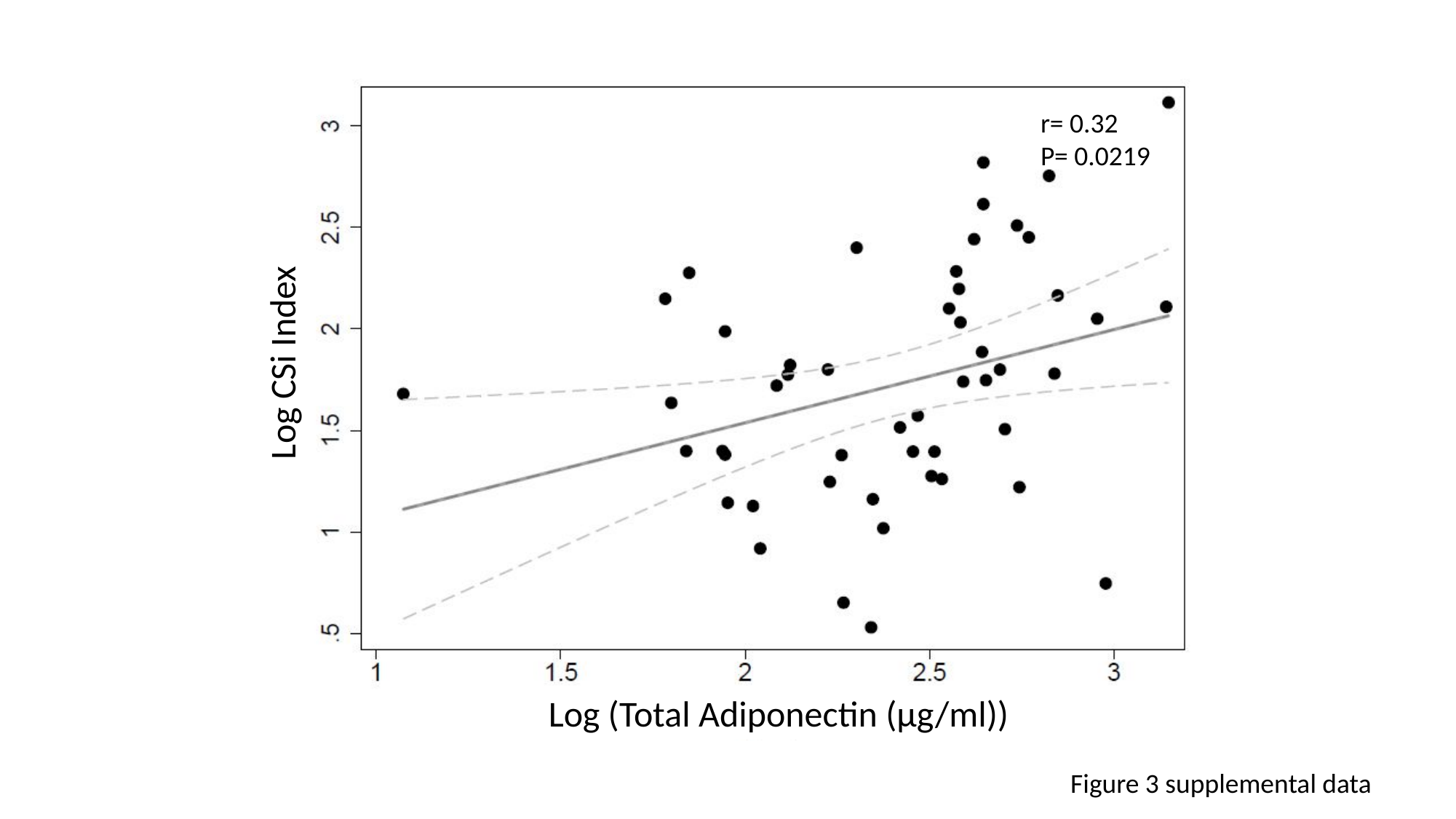

r= 0.32
P= 0.0219
 Log CSi Index
Log (Total Adiponectin (µg/ml))
Figure 3 supplemental data

## Slide 7
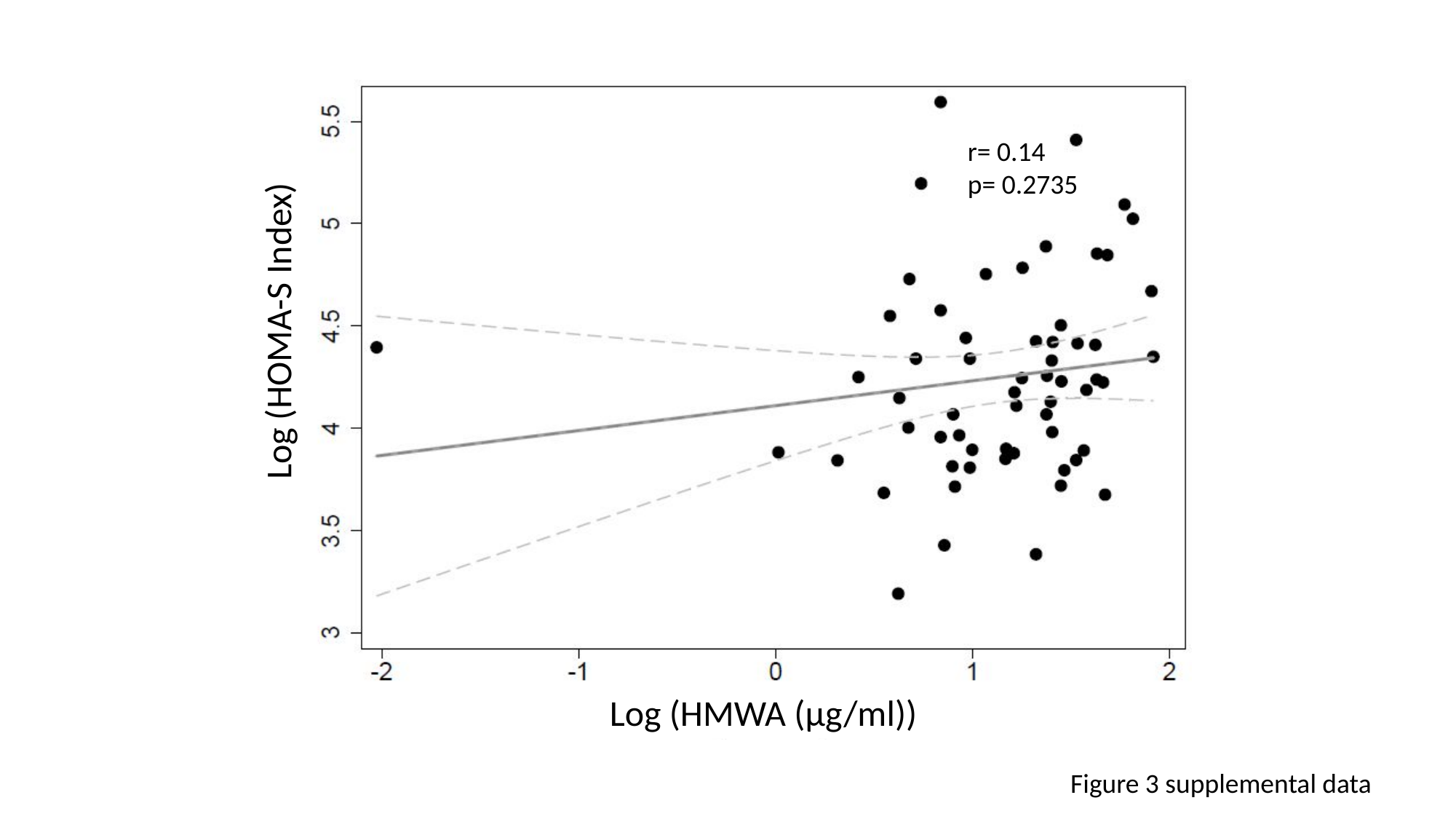

r= 0.14
p= 0.2735
 Log (HOMA-S Index)
Log (HMWA (µg/ml))
Figure 3 supplemental data

## Slide 8
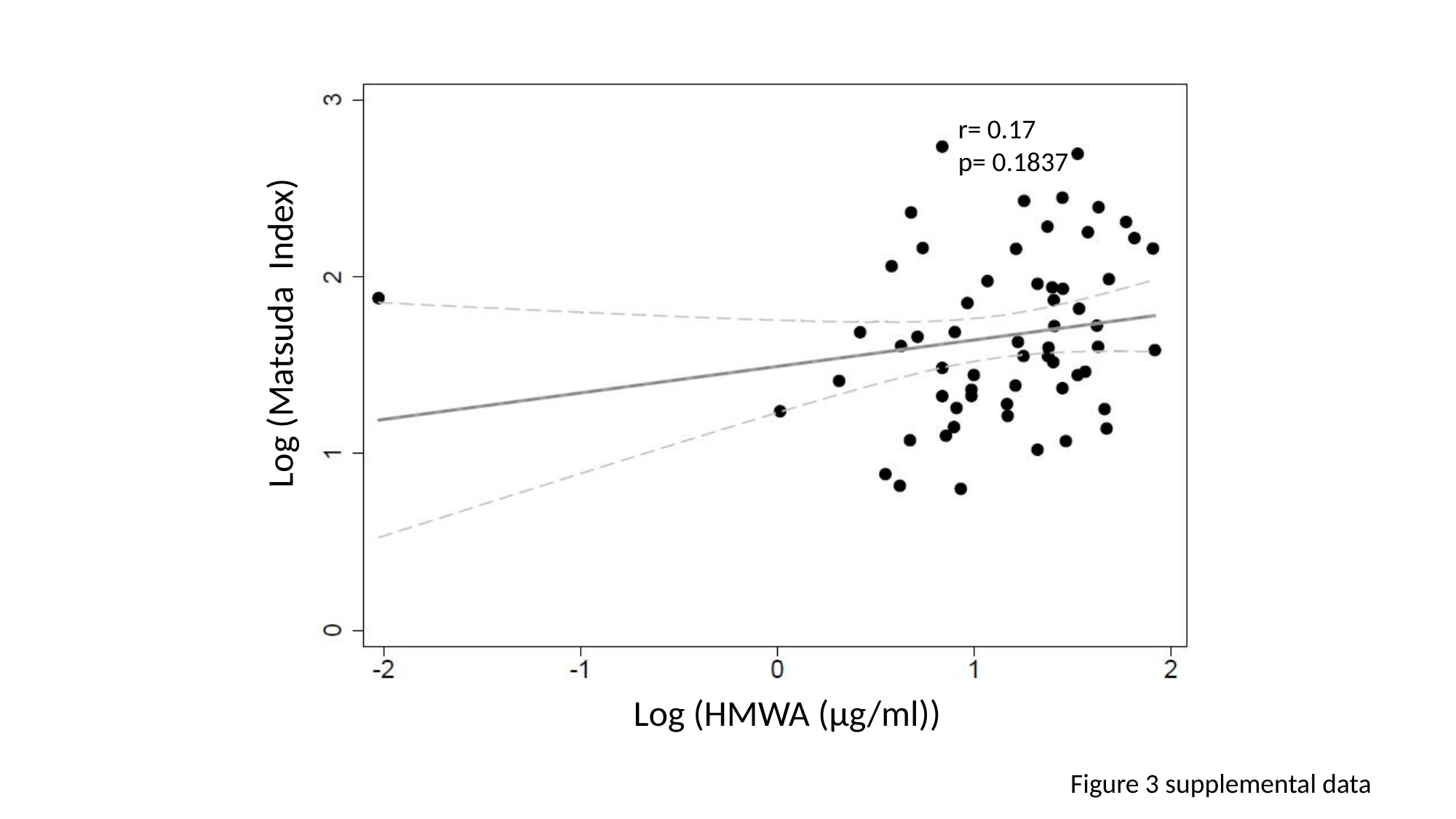

r= 0.17
p= 0.1837
 Log (Matsuda Index)
Log (HMWA (µg/ml))
Figure 3 supplemental data

## Slide 9
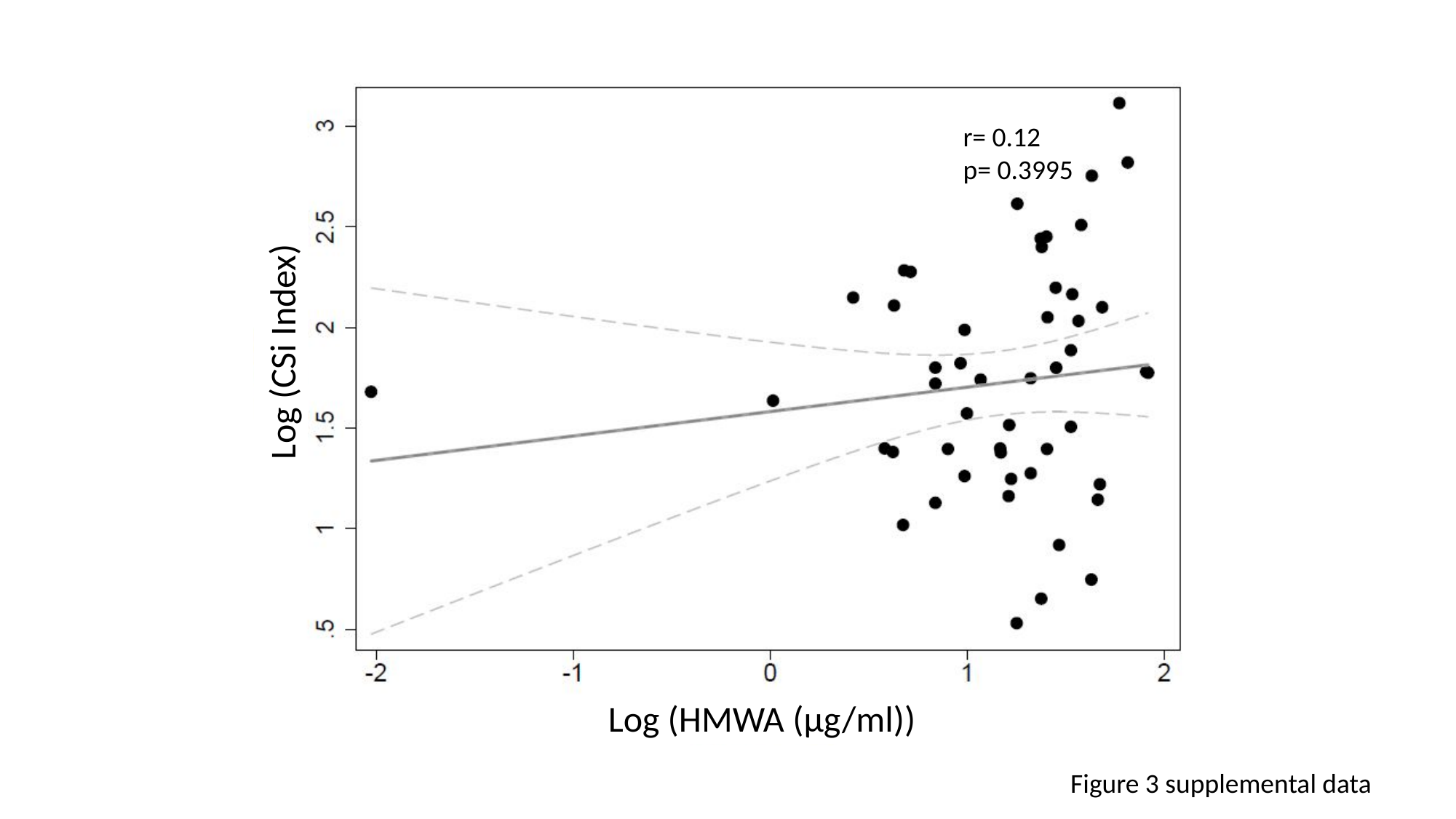

r= 0.12
p= 0.3995
 Log (CSi Index)
Log (HMWA (µg/ml))
Figure 3 supplemental data
