# Supplementary material for: Leptin/Adiponectin Ratios Using Either Total Or High-Molecular-Weight Adiponectin as Biomarkers of Systemic Insulin Sensitivity in Normoglycemic Women
Source: J Diabetes Res. 2017 May 25;2017:9031079. doi: 10.1155/2017/9031079 (PMC5463152; doi:10.1155/2017/9031079)
Supplement: Supplementary file 4 [file 9031079.f4.pptx]

## Slide 1
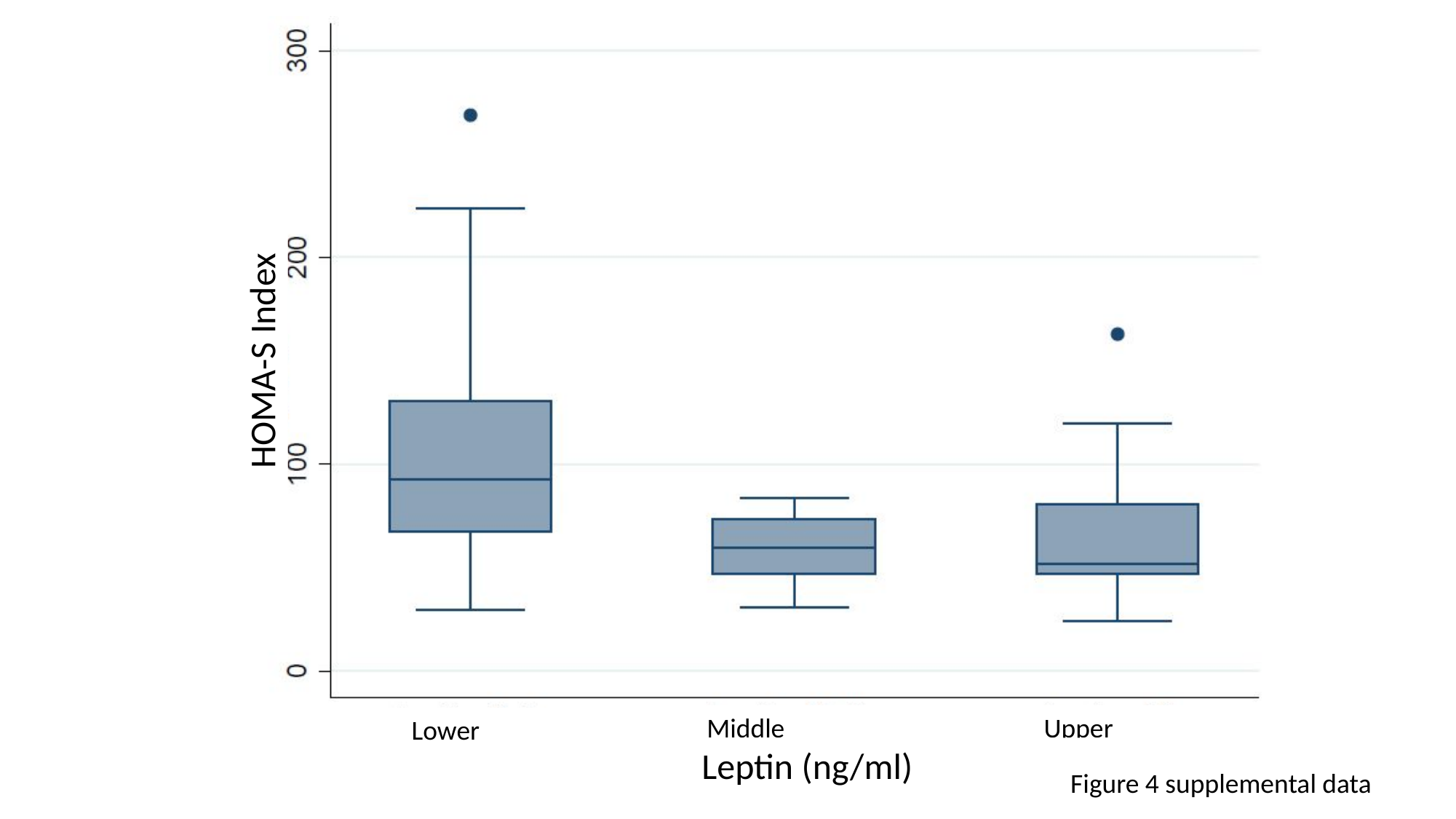

HOMA-S Index
 Middle
 Upper
 Lower
 Leptin (ng/ml)
Figure 4 supplemental data

## Slide 2
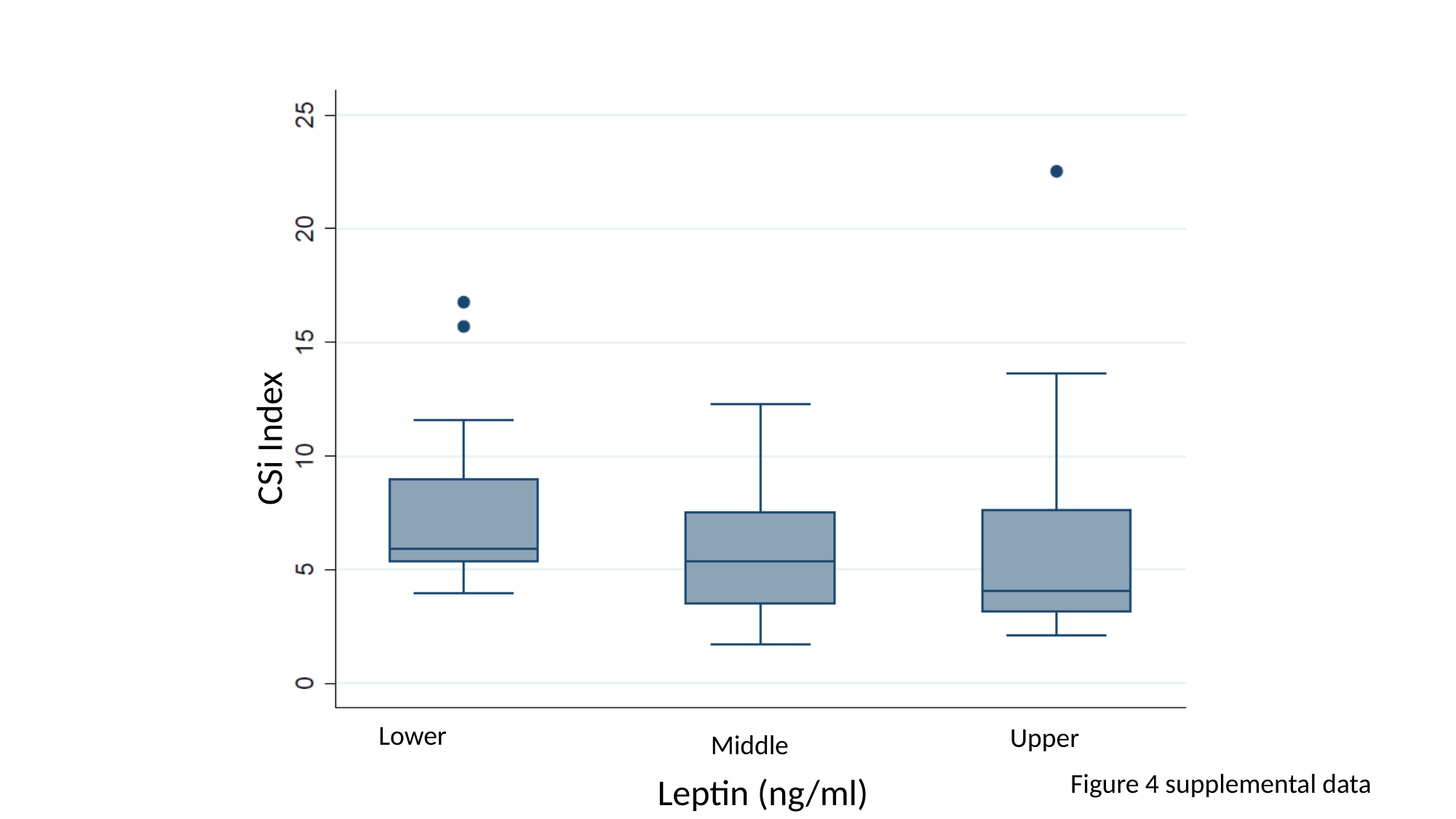

CSi Index
 Middle
Lower
 Upper
Figure 4 supplemental data
 Leptin (ng/ml)

## Slide 3
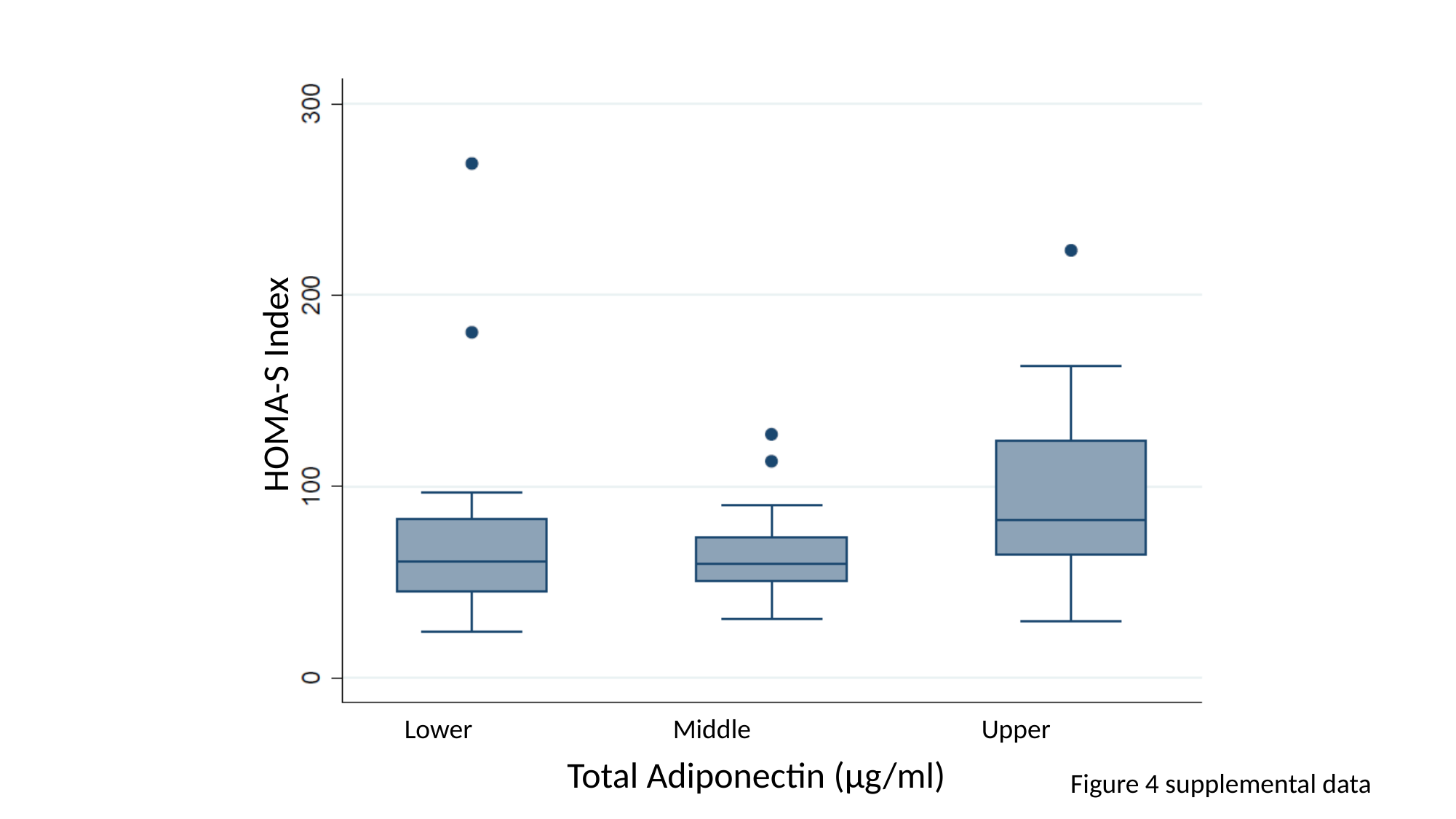

HOMA-S Index
 Middle
 Upper
 Lower
Total Adiponectin (µg/ml)
Figure 4 supplemental data

## Slide 4
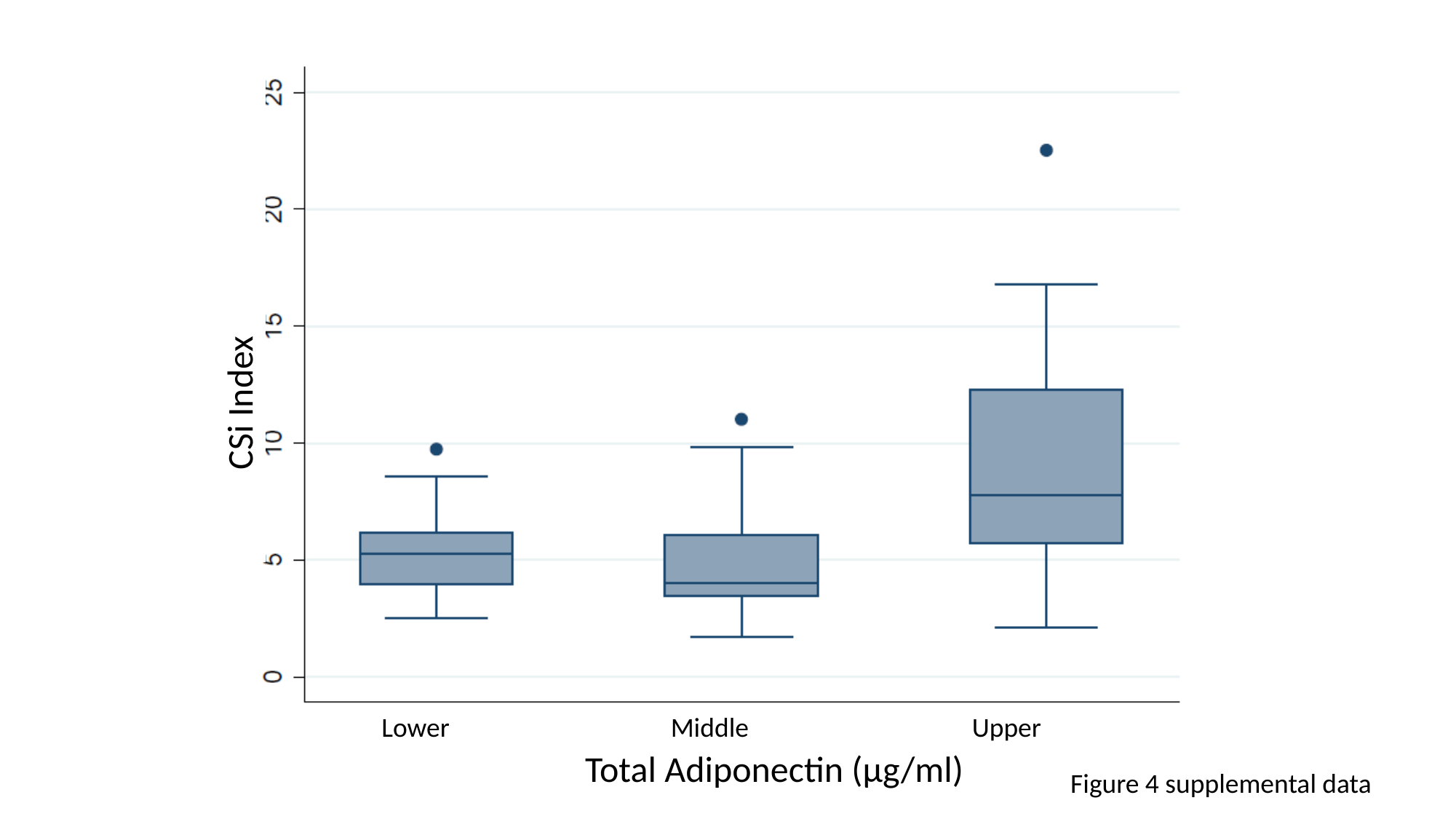

CSi Index
 Lower
 Upper
 Middle
Total Adiponectin (µg/ml)
Figure 4 supplemental data

## Slide 5
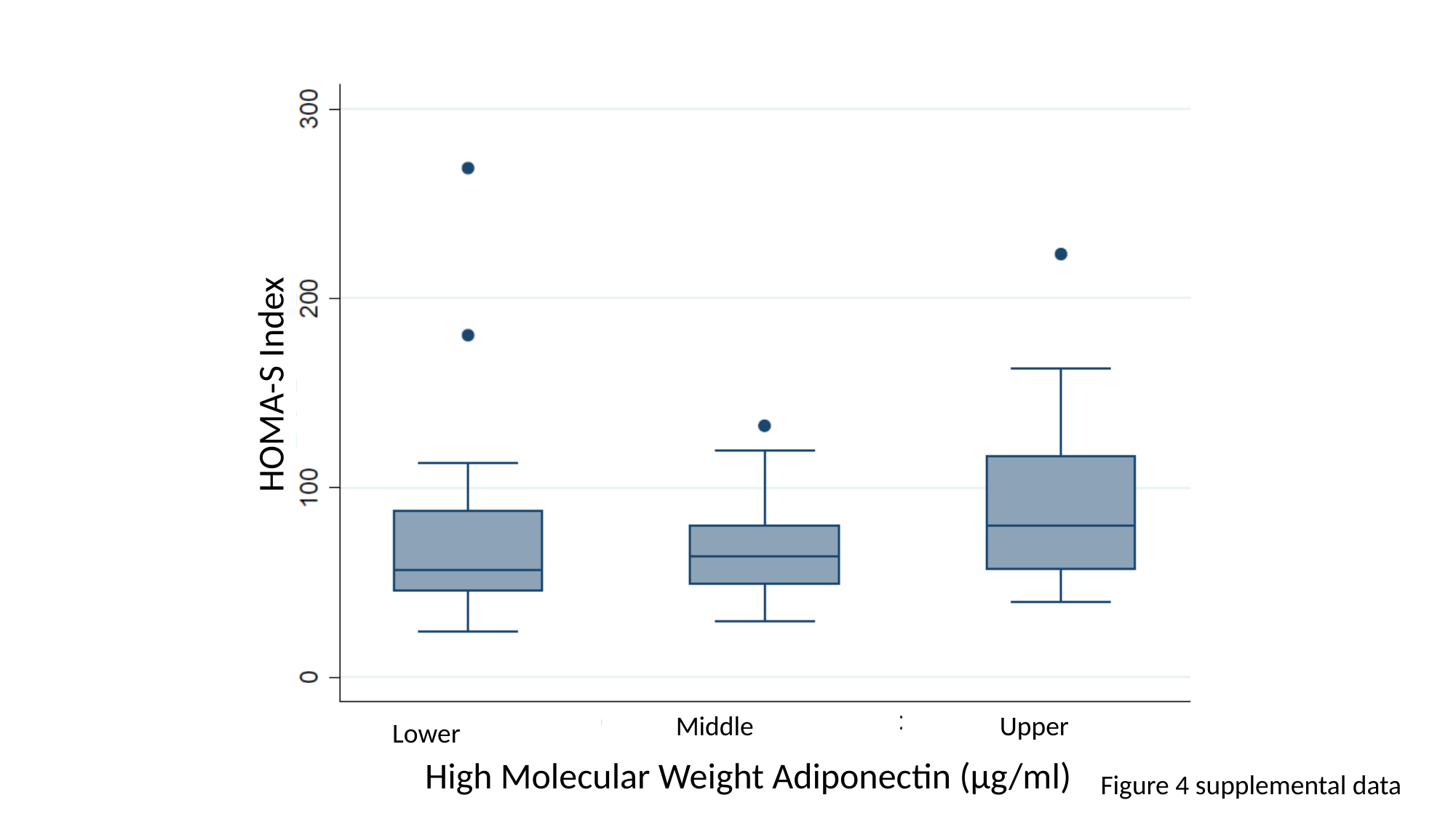

HOMA-S Index
 Middle
 Upper
 Lower
 High Molecular Weight Adiponectin (µg/ml)
Figure 4 supplemental data

## Slide 6
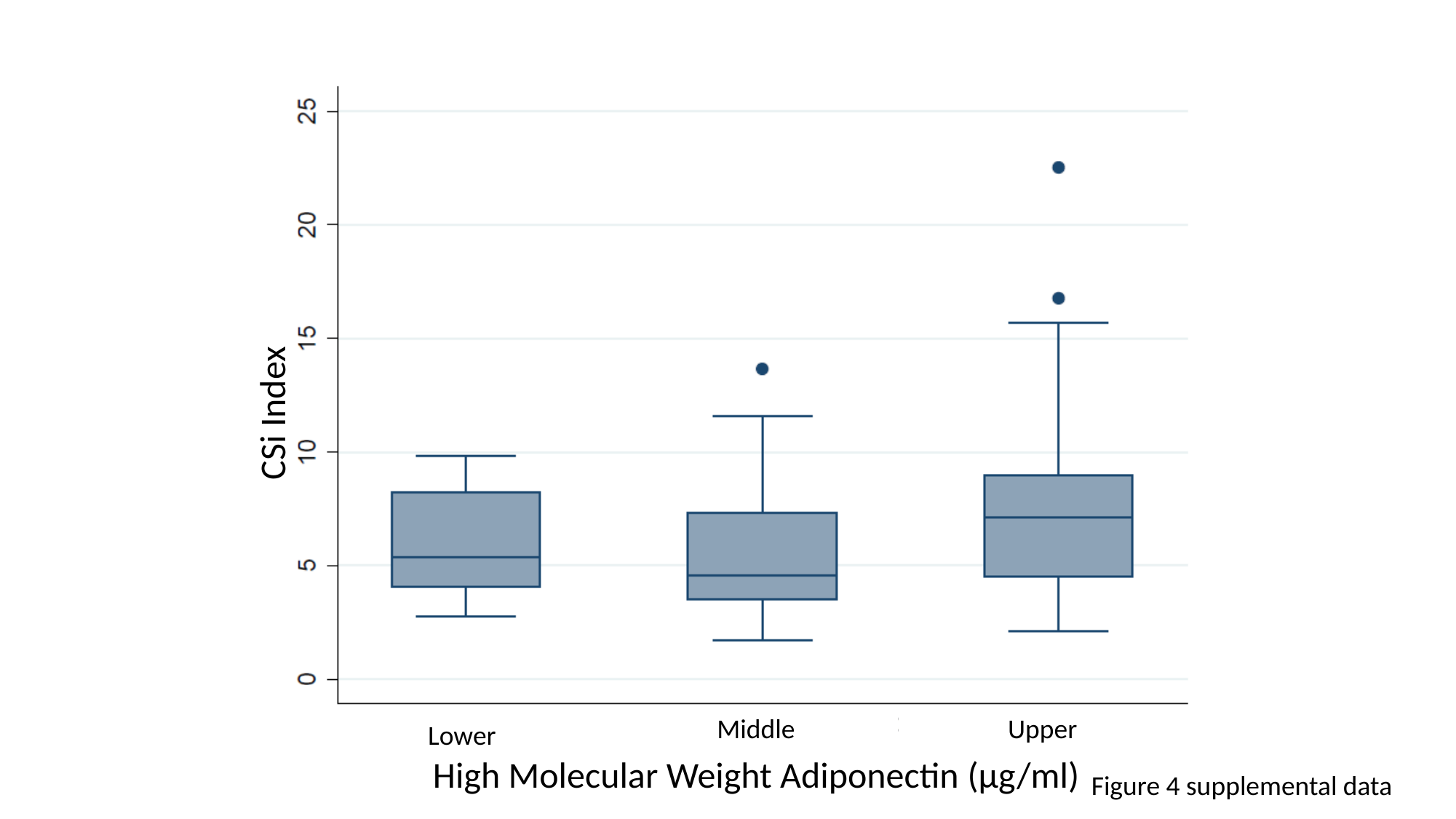

CSi Index
 Middle
 Upper
 Lower
 High Molecular Weight Adiponectin (µg/ml)
Figure 4 supplemental data
