# Supplementary material for: Leptin/Adiponectin Ratios Using Either Total Or High-Molecular-Weight Adiponectin as Biomarkers of Systemic Insulin Sensitivity in Normoglycemic Women
Source: J Diabetes Res. 2017 May 25;2017:9031079. doi: 10.1155/2017/9031079 (PMC5463152; doi:10.1155/2017/9031079)
Supplement: Supplementary file 5 [file 9031079.f5.pptx]

## Slide 1
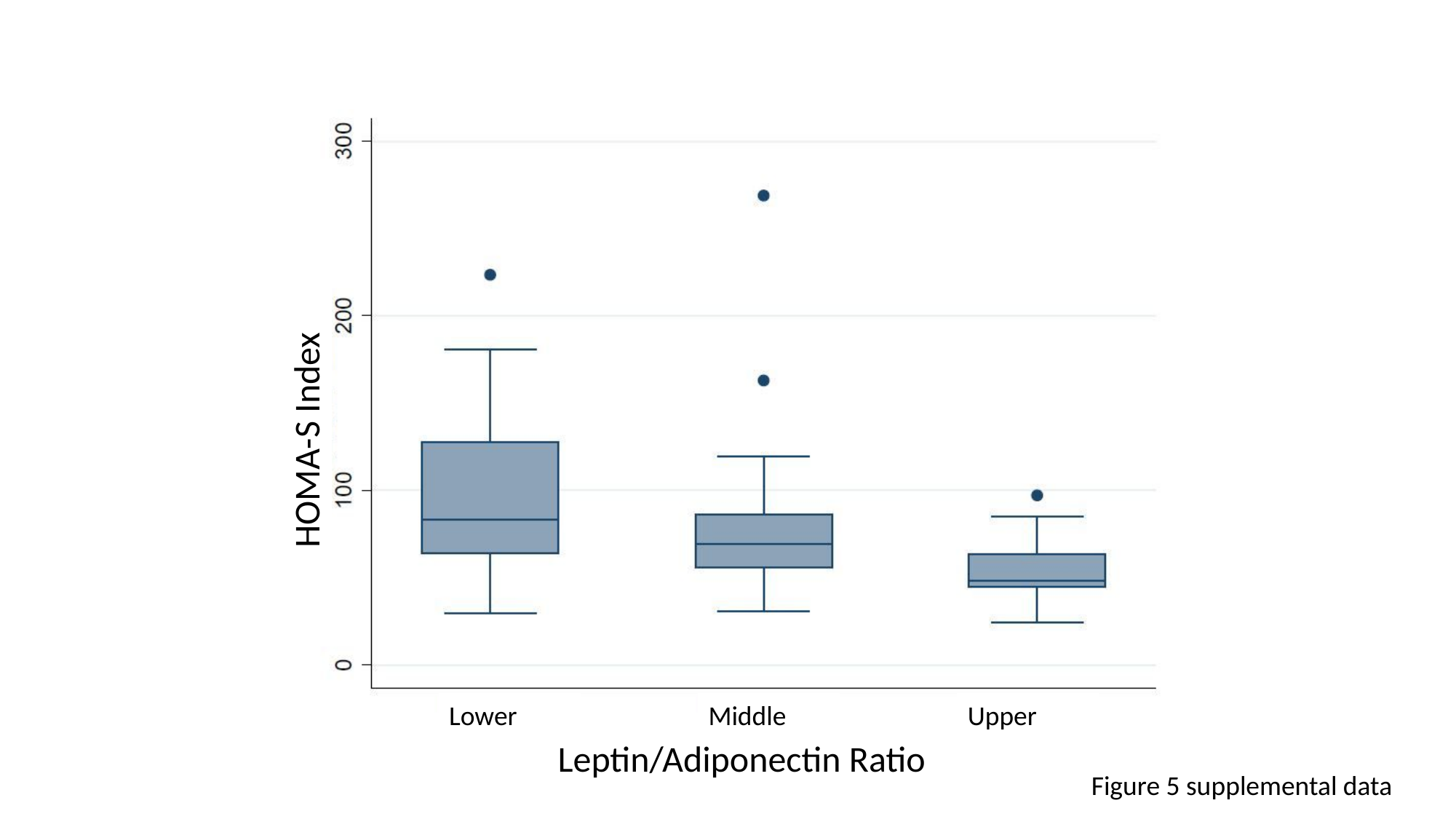

HOMA-S Index
Lower
Middle
Upper
 Leptin/Adiponectin Ratio
Figure 5 supplemental data

## Slide 2
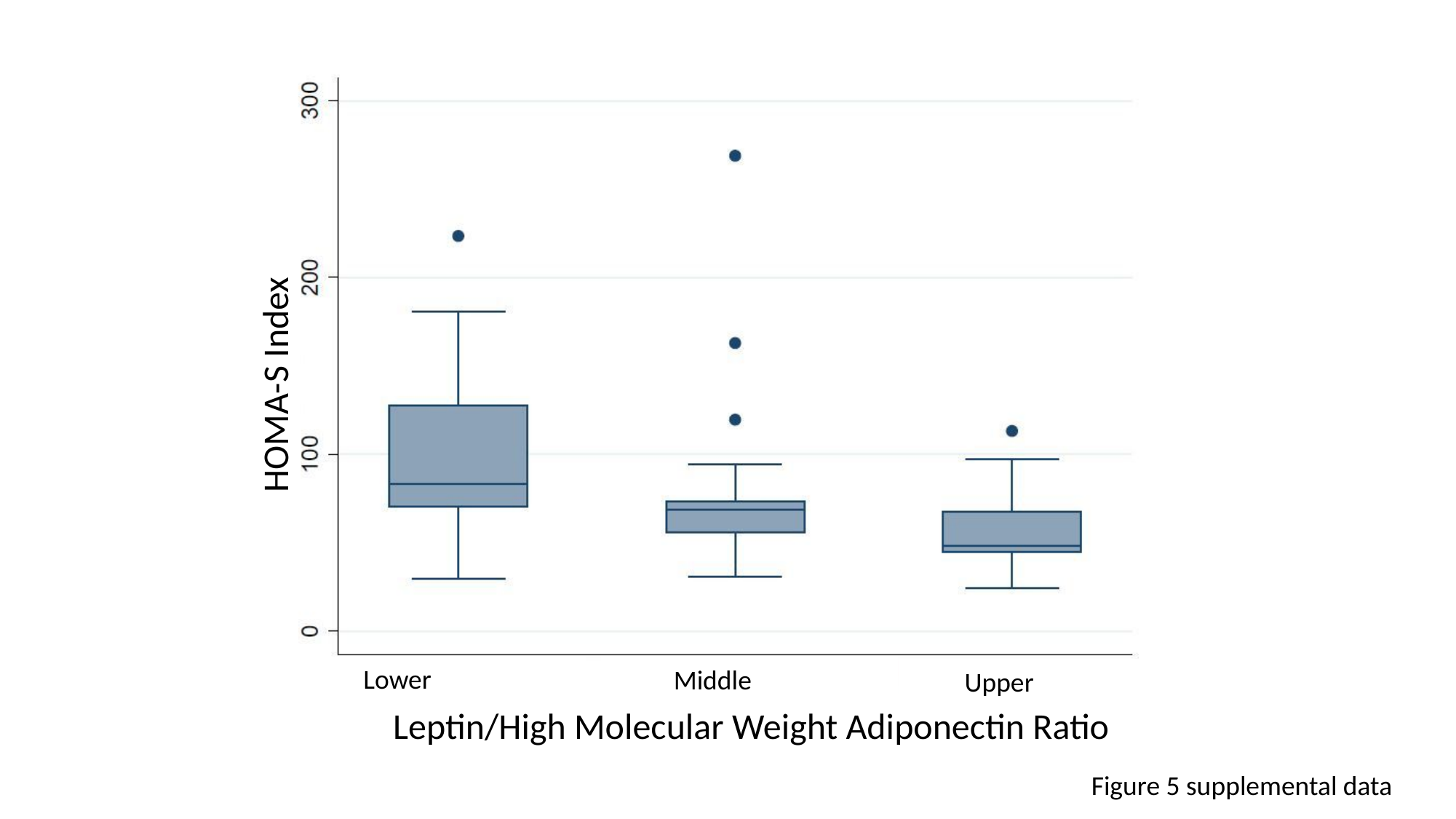

HOMA-S Index
 Lower
 Middle
 Upper
 Leptin/High Molecular Weight Adiponectin Ratio
Figure 5 supplemental data

## Slide 3
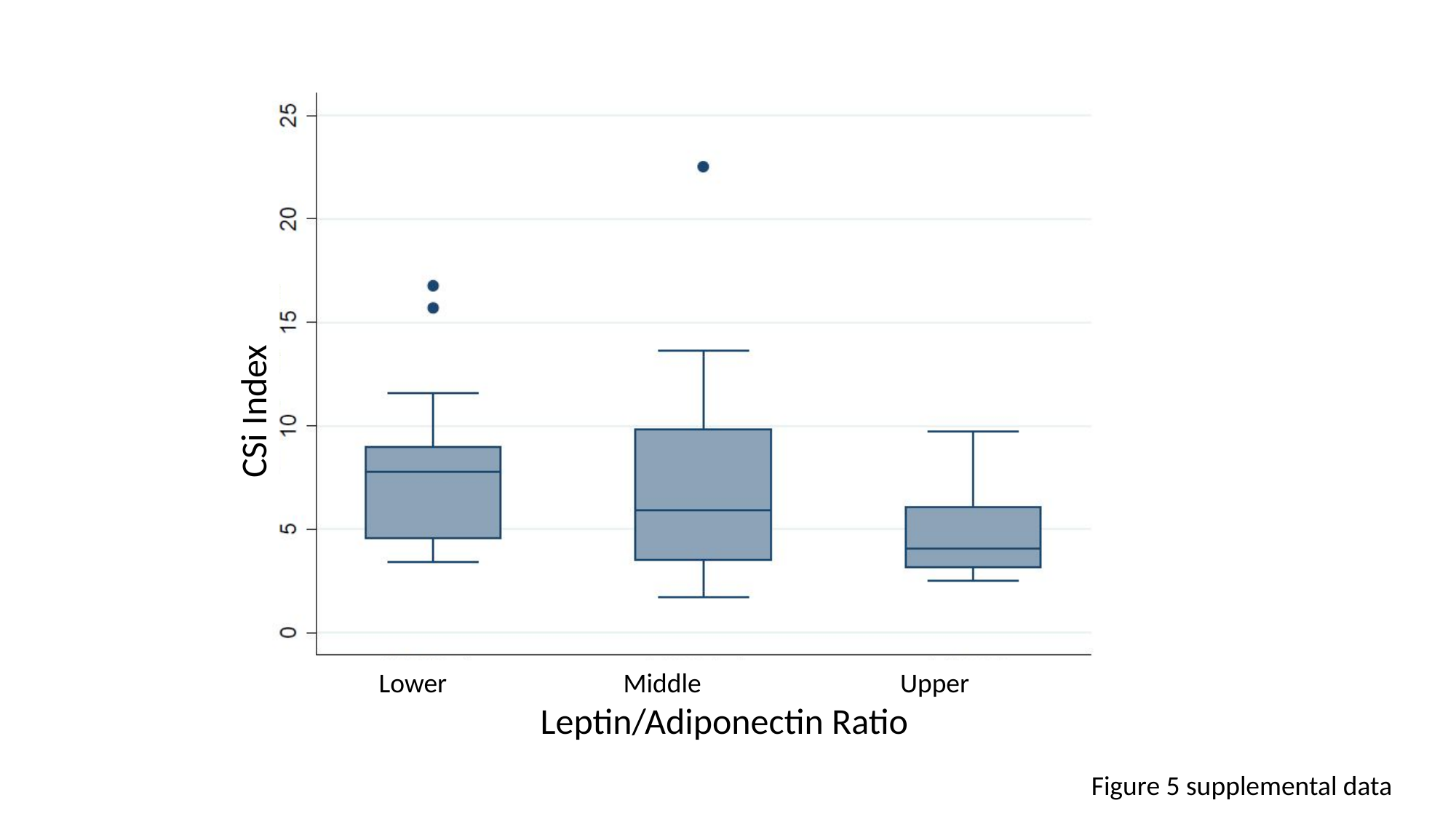

CSi Index
 Upper
 Middle
 Lower
 Leptin/Adiponectin Ratio
Figure 5 supplemental data

## Slide 4
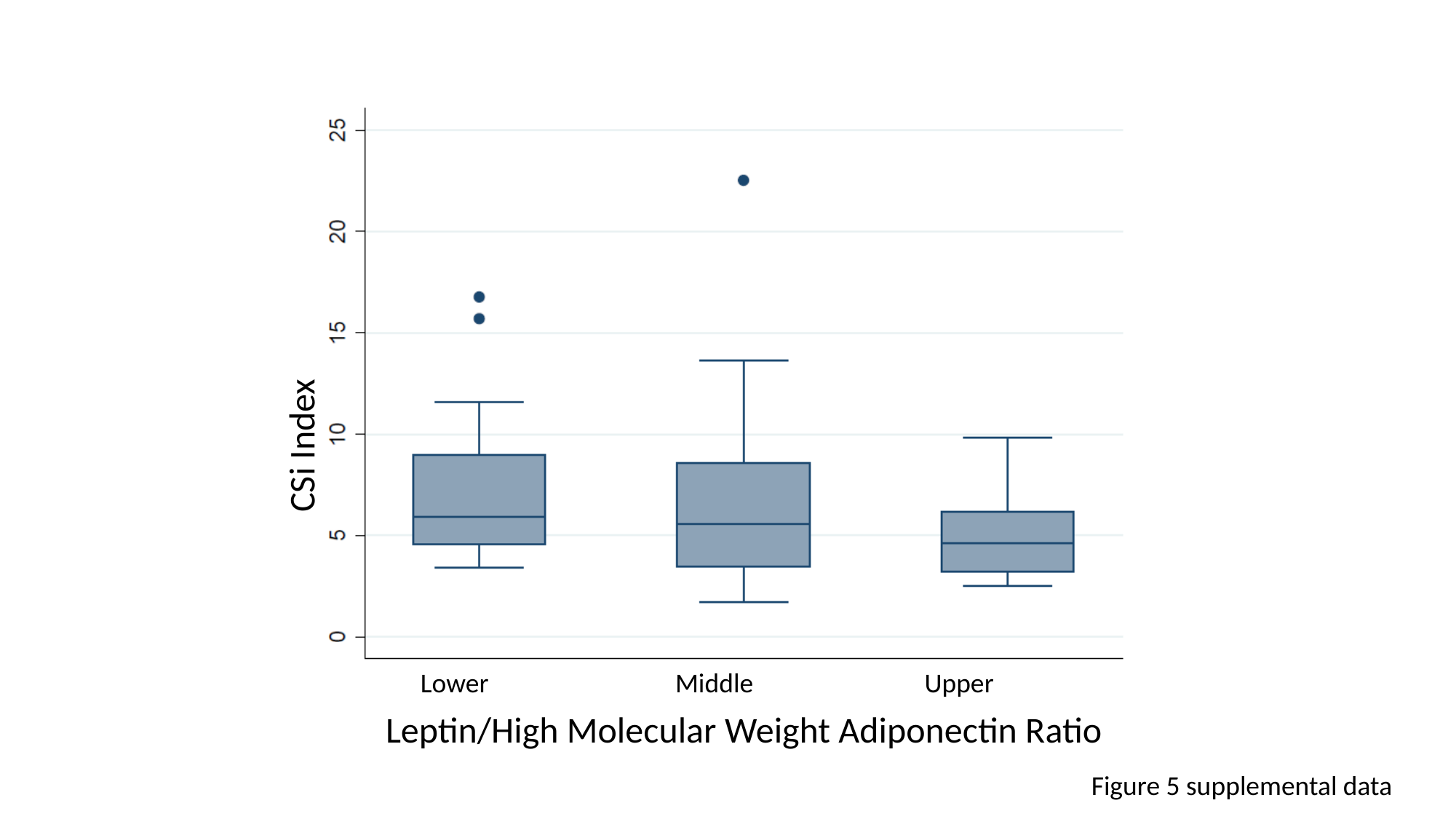

CSi Index
 Lower
 Middle
 Upper
 Leptin/High Molecular Weight Adiponectin Ratio
Figure 5 supplemental data
