# Supplementary material for: Leptin/Adiponectin Ratios Using Either Total Or High-Molecular-Weight Adiponectin as Biomarkers of Systemic Insulin Sensitivity in Normoglycemic Women
Source: J Diabetes Res. 2017 May 25;2017:9031079. doi: 10.1155/2017/9031079 (PMC5463152; doi:10.1155/2017/9031079)
Supplement: Supplementary file 6 [file 9031079.f6.pptx]

## Slide 1
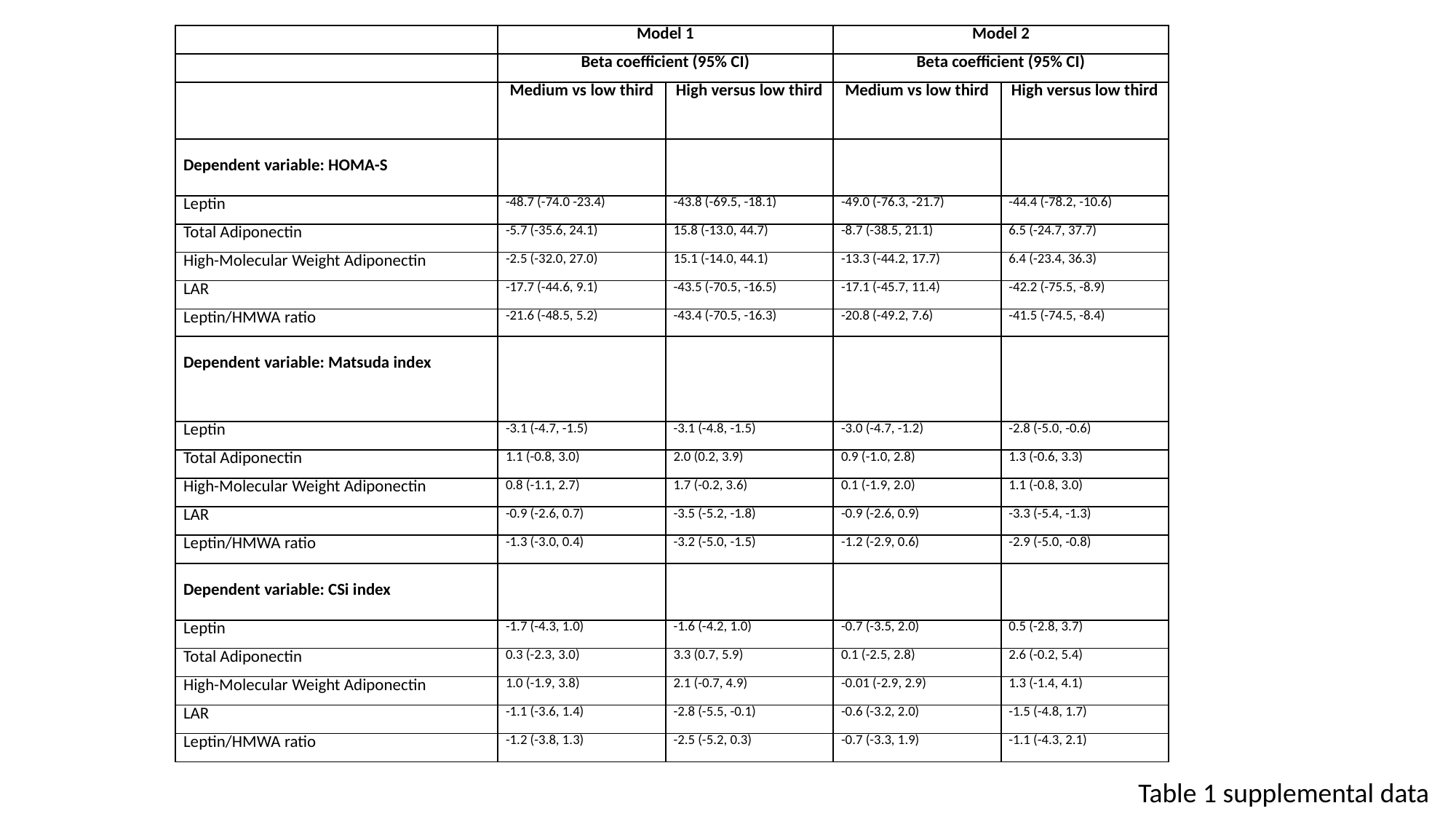

| | Model 1 | | Model 2 | |
| --- | --- | --- | --- | --- |
| | Beta coefficient (95% CI) | | Beta coefficient (95% CI) | |
| | Medium vs low third | High versus low third | Medium vs low third | High versus low third |
| Dependent variable: HOMA-S | | | | |
| Leptin | -48.7 (-74.0 -23.4) | -43.8 (-69.5, -18.1) | -49.0 (-76.3, -21.7) | -44.4 (-78.2, -10.6) |
| Total Adiponectin | -5.7 (-35.6, 24.1) | 15.8 (-13.0, 44.7) | -8.7 (-38.5, 21.1) | 6.5 (-24.7, 37.7) |
| High-Molecular Weight Adiponectin | -2.5 (-32.0, 27.0) | 15.1 (-14.0, 44.1) | -13.3 (-44.2, 17.7) | 6.4 (-23.4, 36.3) |
| LAR | -17.7 (-44.6, 9.1) | -43.5 (-70.5, -16.5) | -17.1 (-45.7, 11.4) | -42.2 (-75.5, -8.9) |
| Leptin/HMWA ratio | -21.6 (-48.5, 5.2) | -43.4 (-70.5, -16.3) | -20.8 (-49.2, 7.6) | -41.5 (-74.5, -8.4) |
| Dependent variable: Matsuda index | | | | |
| Leptin | -3.1 (-4.7, -1.5) | -3.1 (-4.8, -1.5) | -3.0 (-4.7, -1.2) | -2.8 (-5.0, -0.6) |
| Total Adiponectin | 1.1 (-0.8, 3.0) | 2.0 (0.2, 3.9) | 0.9 (-1.0, 2.8) | 1.3 (-0.6, 3.3) |
| High-Molecular Weight Adiponectin | 0.8 (-1.1, 2.7) | 1.7 (-0.2, 3.6) | 0.1 (-1.9, 2.0) | 1.1 (-0.8, 3.0) |
| LAR | -0.9 (-2.6, 0.7) | -3.5 (-5.2, -1.8) | -0.9 (-2.6, 0.9) | -3.3 (-5.4, -1.3) |
| Leptin/HMWA ratio | -1.3 (-3.0, 0.4) | -3.2 (-5.0, -1.5) | -1.2 (-2.9, 0.6) | -2.9 (-5.0, -0.8) |
| Dependent variable: CSi index | | | | |
| Leptin | -1.7 (-4.3, 1.0) | -1.6 (-4.2, 1.0) | -0.7 (-3.5, 2.0) | 0.5 (-2.8, 3.7) |
| Total Adiponectin | 0.3 (-2.3, 3.0) | 3.3 (0.7, 5.9) | 0.1 (-2.5, 2.8) | 2.6 (-0.2, 5.4) |
| High-Molecular Weight Adiponectin | 1.0 (-1.9, 3.8) | 2.1 (-0.7, 4.9) | -0.01 (-2.9, 2.9) | 1.3 (-1.4, 4.1) |
| LAR | -1.1 (-3.6, 1.4) | -2.8 (-5.5, -0.1) | -0.6 (-3.2, 2.0) | -1.5 (-4.8, 1.7) |
| Leptin/HMWA ratio | -1.2 (-3.8, 1.3) | -2.5 (-5.2, 0.3) | -0.7 (-3.3, 1.9) | -1.1 (-4.3, 2.1) |
Table 1 supplemental data
