# Supplementary material for: Leptin/Adiponectin Ratios Using Either Total Or High-Molecular-Weight Adiponectin as Biomarkers of Systemic Insulin Sensitivity in Normoglycemic Women
Source: J Diabetes Res. 2017 May 25;2017:9031079. doi: 10.1155/2017/9031079 (PMC5463152; doi:10.1155/2017/9031079)
Supplement: Supplementary file 7 [file 9031079.f7.pptx]

## Slide 1
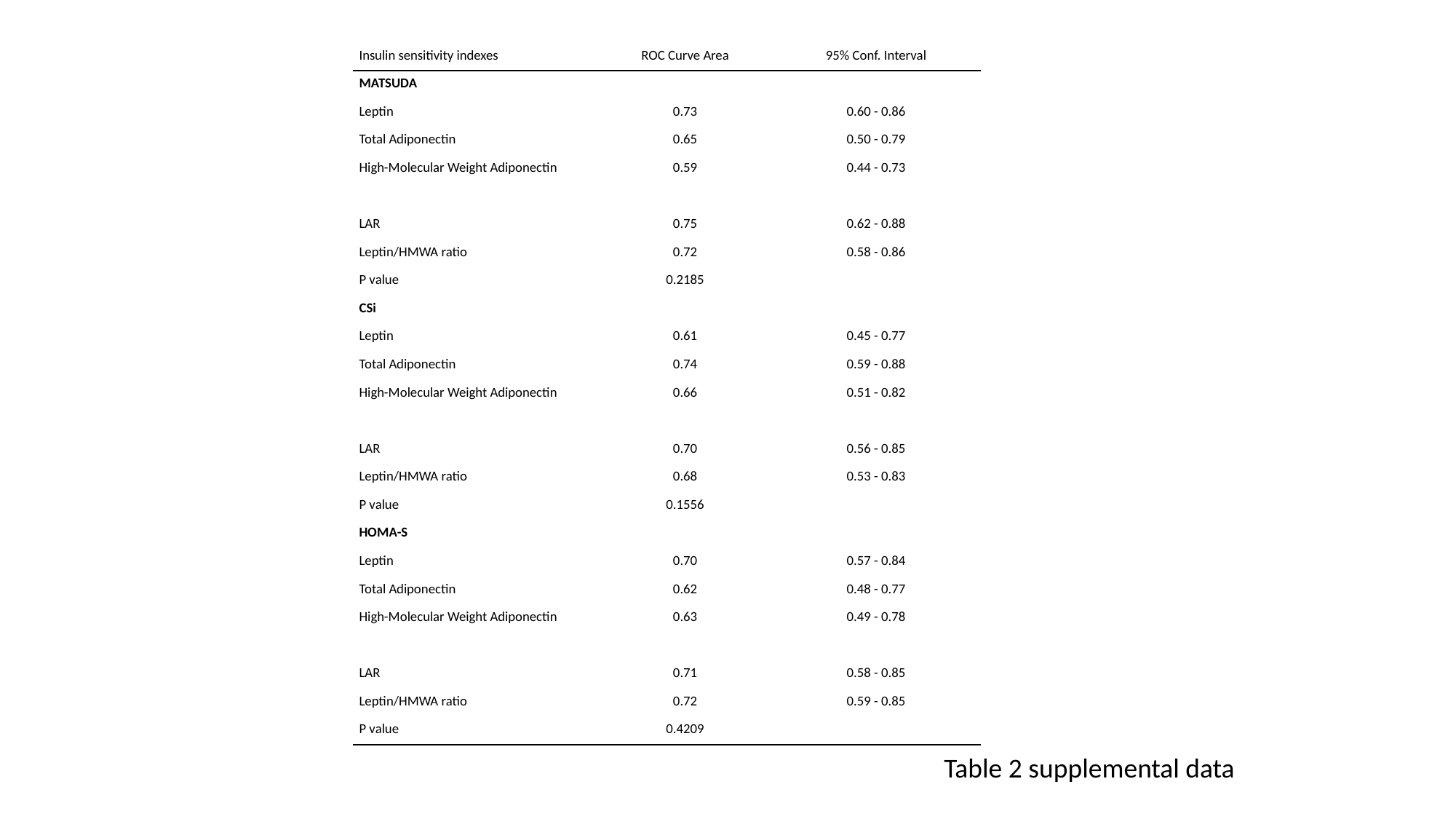

| Insulin sensitivity indexes | ROC Curve Area | 95% Conf. Interval |
| --- | --- | --- |
| MATSUDA | | |
| Leptin | 0.73 | 0.60 - 0.86 |
| Total Adiponectin | 0.65 | 0.50 - 0.79 |
| High-Molecular Weight Adiponectin | 0.59 | 0.44 - 0.73 |
| LAR | 0.75 | 0.62 - 0.88 |
| Leptin/HMWA ratio | 0.72 | 0.58 - 0.86 |
| P value | 0.2185 | |
| CSi | | |
| Leptin | 0.61 | 0.45 - 0.77 |
| Total Adiponectin | 0.74 | 0.59 - 0.88 |
| High-Molecular Weight Adiponectin | 0.66 | 0.51 - 0.82 |
| LAR | 0.70 | 0.56 - 0.85 |
| Leptin/HMWA ratio | 0.68 | 0.53 - 0.83 |
| P value | 0.1556 | |
| HOMA-S | | |
| Leptin | 0.70 | 0.57 - 0.84 |
| Total Adiponectin | 0.62 | 0.48 - 0.77 |
| High-Molecular Weight Adiponectin | 0.63 | 0.49 - 0.78 |
| LAR | 0.71 | 0.58 - 0.85 |
| Leptin/HMWA ratio | 0.72 | 0.59 - 0.85 |
| P value | 0.4209 | |
Table 2 supplemental data
